# Experimental and Numerical Investigation of the Dynamic Characteristics of a Cracked Blisk Under Variable Operating Conditions

**DOI:** 10.3390/ma19173597

**Published:** 2026-08-24

**Authors:** Jiao Wang, Tianci Chen, Longyi Du, Ziyu Tang, Tao Yu, Hong Yuan, Yuehao Zhang

**Affiliations:** 1 School of Electromechanical and Automotive Engineering, Yantai University, Yantai 264005, China; 2 Shandong Center of Technology Innovation for New Energy Vehicle Electric Drive Systems, Yantai 264005, China; 3 School of Intelligent Manufacturing and Automotive Engineering, Guangdong Business and Technology University, Zhaoqing 526040, China; 4 Engineering Training Centre, Yantai University, Yantai 264005, China

**Keywords:** blisk, crack, finite element model, vibration testing, vibration localization

## Abstract

Blisk, as a critical component of aero−engines, is prone to fatigue cracks that can severely impair its dynamic performance and operational safety. This paper develops a finite element model of a blisk with breathing cracks to investigate the effects of crack distribution (adjacent blades vs. separated blades) and blade twist angle on natural frequencies, frequency−veering and mode localization, and response localization under varying operating conditions. Resonance vibration tests are conducted on blisk and cracked blisk specimens using the frequency dwell method to obtain natural frequencies and vibration responses, thereby experimentally validating the numerical model. The results indicate that cracks reduce the global stiffness, leading to a decrease in natural frequencies and modal localization phenomena. Furthermore, in the frequency−domain responses, nonlinear components such as sub−harmonics, ultra−sub−harmonics, and super−harmonics are observed in addition to the harmonics of the excitation frequency. Moreover, the cracked blisk with a 45° blade twist angle exhibits typical nonlinear vibration behaviors and the most pronounced response localization. This study provides a basis for crack monitoring and resonance fatigue warning.

## 1. Introduction

Compared with a tenon–blade–disk, a blisk eliminates the need for tenon connections, significantly reducing engine weight and avoiding fretting wear caused by contact friction at the tenon joint. However, under long−term exposure to high temperatures and high centrifugal forces, the entire compressor blisk is subjected to complex cyclic loads and substantial elastic stress cycling, making it prone to crack failure. According to the data, in the more than 800 flight accidents that occurred in United States Air Force aircraft in the late 1990s, engine failure accounted for 43.5% of the aircraft accidents, and cracks accounted for more than 60%; the above engine failure made it unable to work normally in flight [1].

Researchers worldwide have carried out extensive work on the effect of cracks on the vibration characteristics of blisks through theoretical analysis, numerical simulation, and experimental studies. However, most existing investigations are confined to mistuning, open cracks, or simplified single−sector models of the blisks. Troy et al. [2] validate a new generalized model of mistuning (GMM) for bladed disks. The GMM handles stiffness, damping, and geometric mistuning uniformly. Comparison with finite element results shows that the GMM accurately predicts dynamic responses across different mistuning types, offering an efficient tool for mistuning analysis. Sun et al. [3] established a dynamic model that comprehensively considers blade–disk coupling, inter−stage stiffness, and mistuning disturbances, which can accurately characterize the free and forced vibration behaviors of the system. Hou et al. [4] used lumped−mass beams with root cracks, computed stiffness loss via the flexibility matrix, and derived the cracked blade–disk dynamic equations. Fang et al. [5] evaluated blade stiffness loss via fracture mechanics, modeled the bladed disk as spring−coupled cantilever beams, and derived analytical free/forced vibration solutions for the cracked disk. Kuang et al. [6] modeled a cracked blade as a torsional−spring two−span beam via Euler–Bernoulli and Galerkin methods. Crack flexibility was obtained from fracture mechanics. The shrouded bladed disk equations were derived, and free/harmonic responses were analyzed. Wang et al. [7] defined the mode localization factor, providing a quantitative method to evaluate the degree of vibration mode localization in detuned blade–disk structure. Yang et al. [8] have established a variety of working conditions, and the results show that the model can theoretically explain the general coupling mechanism, and it is found that the installation angle, the coupling stiffness of the wafer, and the position of the disk have a great influence on the coupling. Wang et al. [9] studied the relationship between the frequency steering of the blisk structure and the energy of each sub−mode through the lumped parameter model. Xu et al. [10] proposed a new method using hard coating to control the detuning level of blades, and the results show that this method can effectively control the detuning level. Matteo et al. [11] developed a computational scheme for viscoelastic damping layers on rotating structures and applied it to a fan−bladed disk. Numerical and experimental comparisons validated the method’s effectiveness. Nan et al. [12] adopted numerical simulation combined with theoretical analysis and experimental verification. They established a dynamic model of the bladed disk based on cyclic symmetry theory, used ANSYS 2024R1 to analyze the influence of crack location on natural frequencies, vibration responses, and fatigue life, and corrected the damping ratio through vibration experiments. Wu et al. [13] built an analytical model for a cracked−blade−flexible−disk−elastic−support system using plate/beam theories and the strain energy release method, and studied the effects of crack depth and location. Finite element analysis (FEA) and experiments confirmed model accuracy. Wang et al. [14] investigated the effects of cracks and mistuning on the vibration response and localization of an impeller system. Their results indicate that both factors can induce significant vibration responses, concentrated vibration energy, and pronounced vibration localization. Yan et al. [15] conducted a crack growth analysis on the final−stage wheel of a steam turbine, examining the stress distribution and crack propagation at the rim location where stress concentration is most severe. Based on engine performance tests, Zhang et al. [16] analyzed the vibration data of the exhaust casing and found that when the crack length ratio is below 0.3, the overall machine vibration exhibits low sensitivity to blade–disk mistuning. Li et al. [17] investigated the mechanical behavior of blade cracks in a K417 alloy cast monolithic blade–disk during test runs, and identified that the primary cause of cracking was resonance induced by the guide vanes. Wang et al. [18] established and experimentally validated a shell element model for an elastically supported cracked disk, uncovering the support–vibration coupling and systematically examining the influences of crack length, circumferential position, and radial position on vibration modes. Wang et al. [19] combined resonance experiments and theoretical modeling, established shell/beam element models with a breathing crack spring, used penalty coupling and mode reduction, and validated via ANSYS and crack propagation tests. Wang et al. [20] used FE simulation with tenon–mortise contact to study blade tip variations in cracked turbine disks, examining four crack types and quantifying the effects of crack geometry (length, width, radial position, number) on tip parameters. Wang et al. [21] investigated the effects of multiple cracks on the forced response of centrifugal impellers and proposed a reduced−order modeling strategy based on the finite element method and hybrid interface component mode synthesis (CMS). They systematically analyzed the natural frequency shifts, nonlinear forced responses, and vibration localization characteristics under various crack lengths and relative positions. Chiu et al. [22] combined theoretical and numerical methods to study the influence of blade cracks and temperature fields on thermoelastic vibrations of a flexible disk rotor system. Using the assumed mode method, they developed a five−field (shaft–disk–blade–heat–crack, SDBHC) coupled model. Modal analysis was conducted in ANSYS, and wavelet packet transform was applied for multi−scale frequency domain decomposition to verify the predictions. Wu et al. [23] studied the shaft–disk−cracked blade coupling system, examining the effects of gravity, rotor unbalance, and aerodynamic loads on blade tip vibration, as well as the influences of crack depth and location. Li et al. [24] simulated a cracked multi−stage shaft–disk–blade assembly via finite element method (FEM). Stress intensity factors were computed, and the effects of edge and mid−span cracks on displacement, stress intensity, and vibration under centrifugal and aerodynamic loads were analyzed. Yang et al. [25] explored the vibration response of a blade–disk–shaft system with breathing cracks and aerodynamic forces. The cracked blade was found to vibrate differently from other blades in the same disk, inducing a mistuning effect. Zhang et al. [26] modeled a shaft–disk–blade system with breathing cracks, solved for tip clearance via Galerkin and Newmark−β, and validated a 3D clearance difference vector by fiber optic experiments for crack localization and severity detection. Yang et al. [27] established a dynamic model of a shaft–disk–blade coupled system based on continuum theory and Lagrange’s equations. The effects of crack parameters, operating conditions, and structural parameters on the coupled vibration response were systematically investigated. Wei et al. [28] studied nonlinear dynamics of a flexible blade–rotor−casing system with breathing cracks and rub−impact. Crack−induced asymmetry generated modulation frequencies, and super−harmonic resonances were transmitted to rotor vibrations through coupling. Yang et al. [29] studied how blade cracks and operating conditions affect coupled vibration in multi−stage bladed disk–rotor systems. Cracks cause notable mistuning and vibration localization, and cracked blades carry much higher vibration energy than uncracked ones in the same disk.

However, the combined effects of breathing crack distributions and blade twist angles on the vibration localization and nonlinear dynamic responses of blisks remain insufficiently explored. To address these issues, this paper establishes full FE models of an aero−engine blisk to systematically investigate their coupled influences, with numerical predictions validated through resonance vibration testing.

## 2. Vibration Testing and Numerical Simulation of a Blisk Specimen

### 2.1. Vibration Testing of the Blisk Specimen

**Figure 1:**
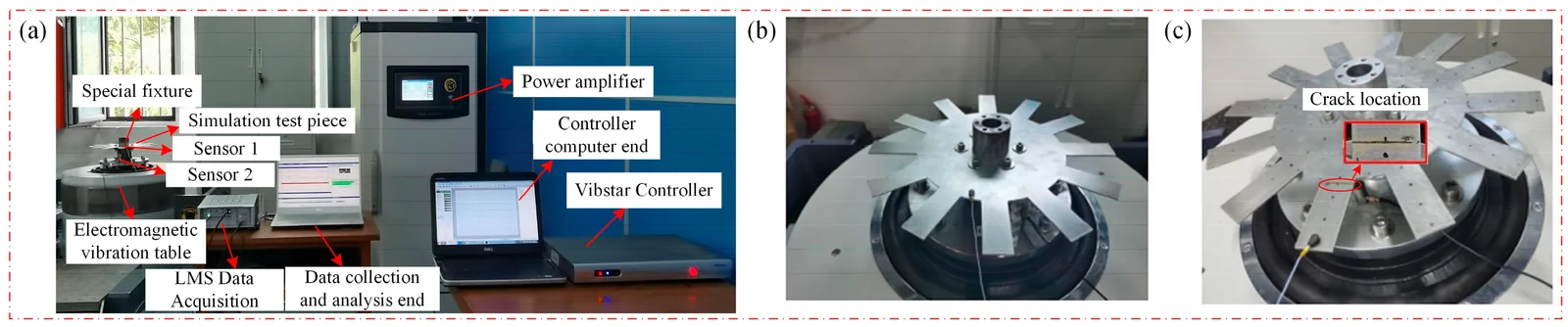
Test platform and blisk specimens: (**a**) test platform, (**b**) intact blisk, (**c**) blisk with 90% chord–width cracks.

**Figure 2:**
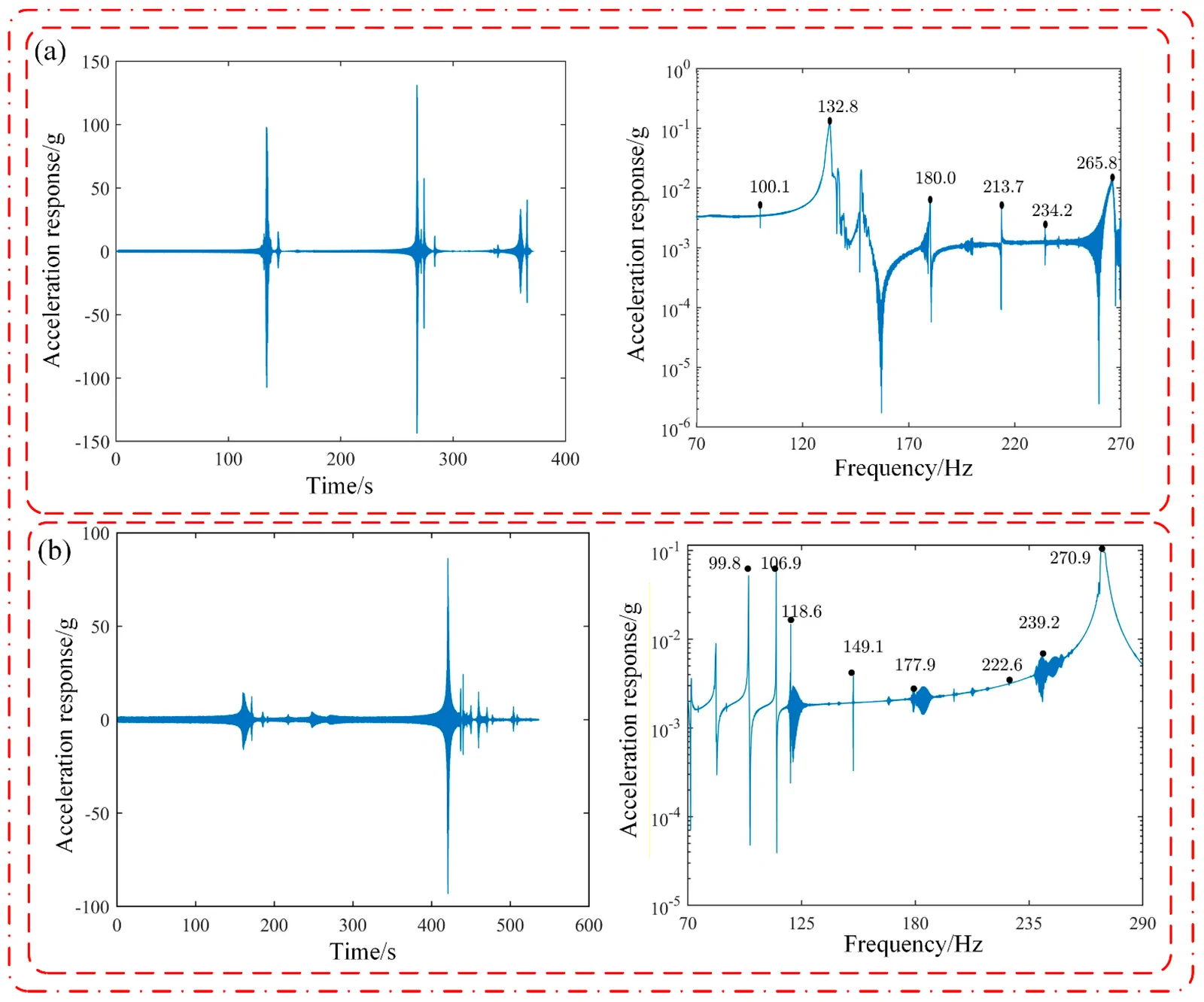
Measured sweep acceleration responses: (**a**) intact blisk and (**b**) cracked blisk.

An experimental test rig was developed to evaluate the vibration characteristics of an integral blisk specimen, as shown in Figure 1a. The main considerations for using a pre−cracked blisk in vibration testing are as follows: (1) excitation feasibility: the blisk specimen can be more readily excited in shaker tests; (2) material limitations: the high yield strength of the titanium alloy makes the initiation of natural fatigue cracks extremely difficult and time−consuming; and (3) resource efficiency: resonance fatigue testing on an actual blisk is both time−consuming and costly. To overcome these challenges and reduce the associated costs, this study adopts a wire−cut pre−cracked specimen to efficiently validate the numerical method. The specimen was mounted on an electrodynamic shaker via a dedicated fixture. Two accelerometers were used in the testing setup. Sensor 1 (PCB352C22; PCB Piezotronics, Inc., Depew, NY, USA) was attached to the disk of the specimen, while Sensor 2 (PCB353B03; PCB Piezotronics, Inc., Depew, NY, USA) was mounted on the shaker to provide closed−loop feedback to the controller, power amplifier, and shaker system. According to the modal analysis results, the eighth−order mode shape exhibits the maximum stress at the blade root. Preliminary finite−element modal analysis yielding von Mises stress results is only used as pre−test planning guidance for estimating potential high−stress regions near the blade root. A detailed discussion of the modal analysis results is provided in Section 2.2. Therefore, a cracked blisk was prepared by wire cutting at the blade root, as shown in Figure 1c. A linear sweep excitation was applied, and the resulting vibration signals were acquired using an LMS vibration measurement system. The resonance method was utilized to determine the natural frequencies over the frequency range of 50 to 1000 Hz. The acquired data served as a reference for the subsequent sweep excitation tests. Time−domain and frequency−domain acceleration responses of the intact and cracked blisks are presented in Figure 2. The results show that the crack reduces the structural stiffness, thereby decreasing the natural frequencies.

**Figure 3:**
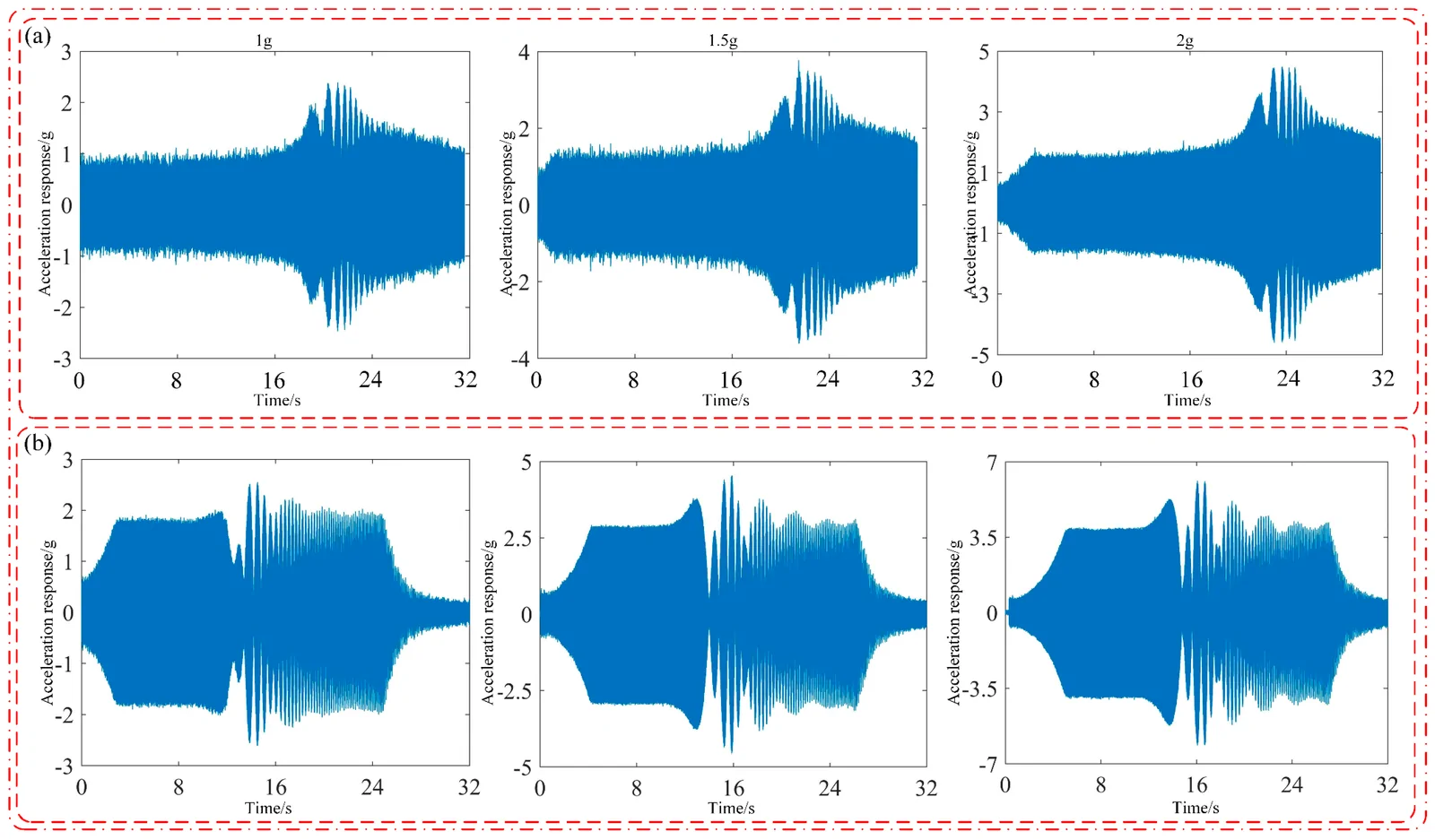
Measured time−domain responses under 1 g, 1.5 g, and 2 g excitations: (**a**) intact blisk and (**b**) cracked blisk.

**Figure 4:**
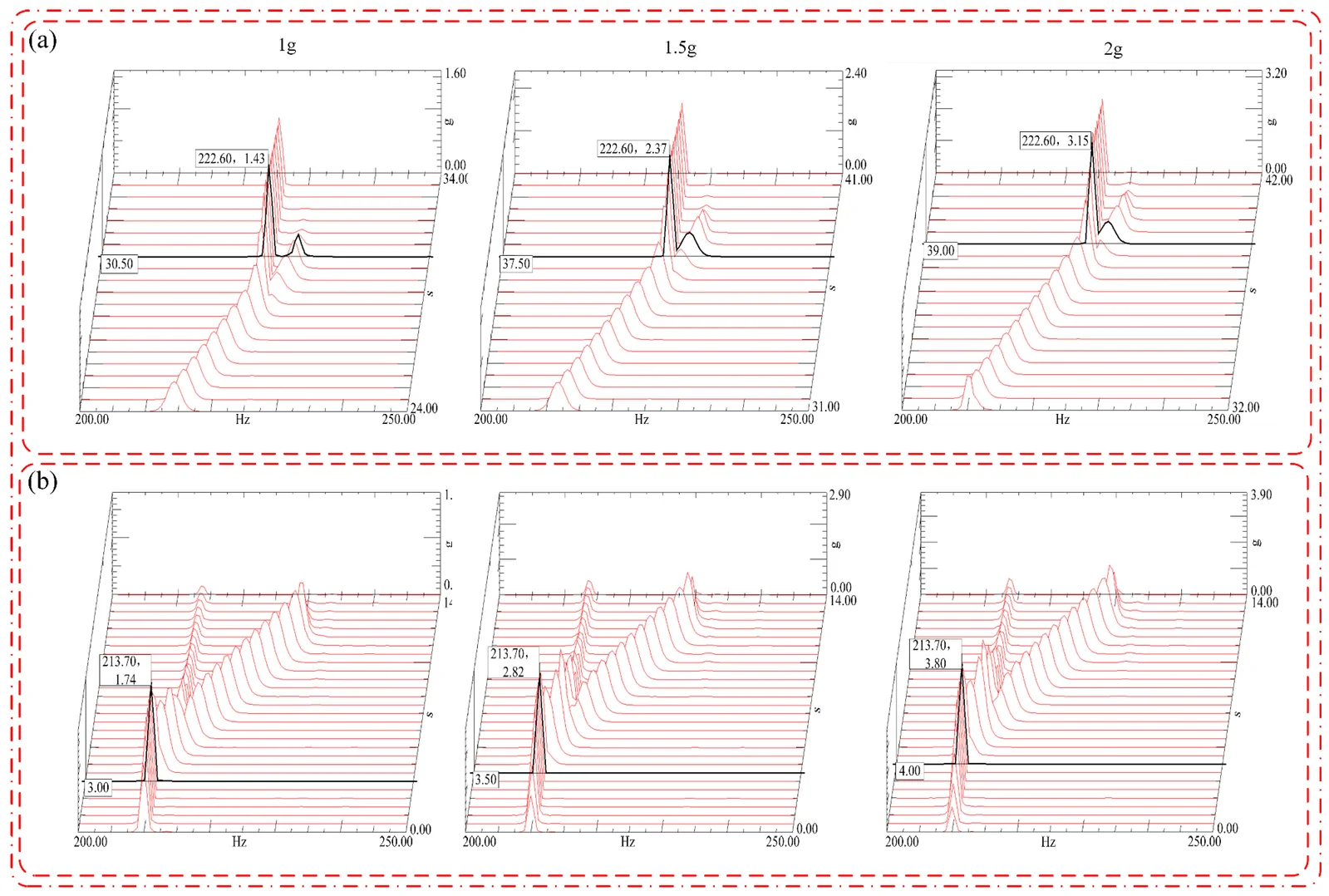
Three–dimensional waterfall plot of the blisk vibration response: (**a**) blisk and (**b**) cracked blisk.

Subsequently, the eighth−order natural frequency was adopted as the excitation frequency, with the associated modal frequency band defined as 210–230 Hz. Sweep frequency excitation tests were then performed within this band at three acceleration levels (1 g, 1.5 g, and 2 g). Here, g denotes the gravitational acceleration (1 g = 9.81 m/s^2^). The corresponding time−domain acceleration responses and three−dimensional waterfall plots, obtained from the intact and cracked blisk specimens under each excitation level, are shown in Figure 3 and Figure 4, respectively.

As shown in Figure 3, during the 0–16 s period, the response amplitude of the intact blisk increases slowly, while the cracked blisk exhibits obvious fluctuations at an earlier stage. During the 16–28 s period, both specimens show intensive vibrations, but the cracked one has significantly higher peak amplitudes, waveform distortion, and impact spike intensity than the intact one. During the 28–32 s period, the amplitude of the intact blisk decreases smoothly, whereas the cracked blisk decays at a faster rate. Overall, the vibration response intensifies with increasing excitation force. The response amplitudes of the intact blisk under excitation forces of 1 g, 1.5 g, and 2 g are 2.39 g, 3.78 g, and 4.49 g, respectively, while those of the cracked blisk are 2.56 g, 4.54 g, and 6.13 g, respectively.

It can be observed from the 3D waterfall diagram in Figure 4 that the presence of cracks reduces the natural frequency and increases the vibration amplitude of the blisk, while the vibration response increases progressively with the rise in excitation force. Overall, the vibration response intensifies with increasing excitation force. The response amplitudes of the intact blisk under excitation forces of 1 g, 1.5 g, and 2 g are 1.43 g, 2.37 g, and 3.15 g, respectively, while those of the cracked blisk are 1.74 g, 2.82 g, and 3.80 g, respectively.

### 2.2. Finite Element Simulation of the Blisk Specimen

**Figure 5:**
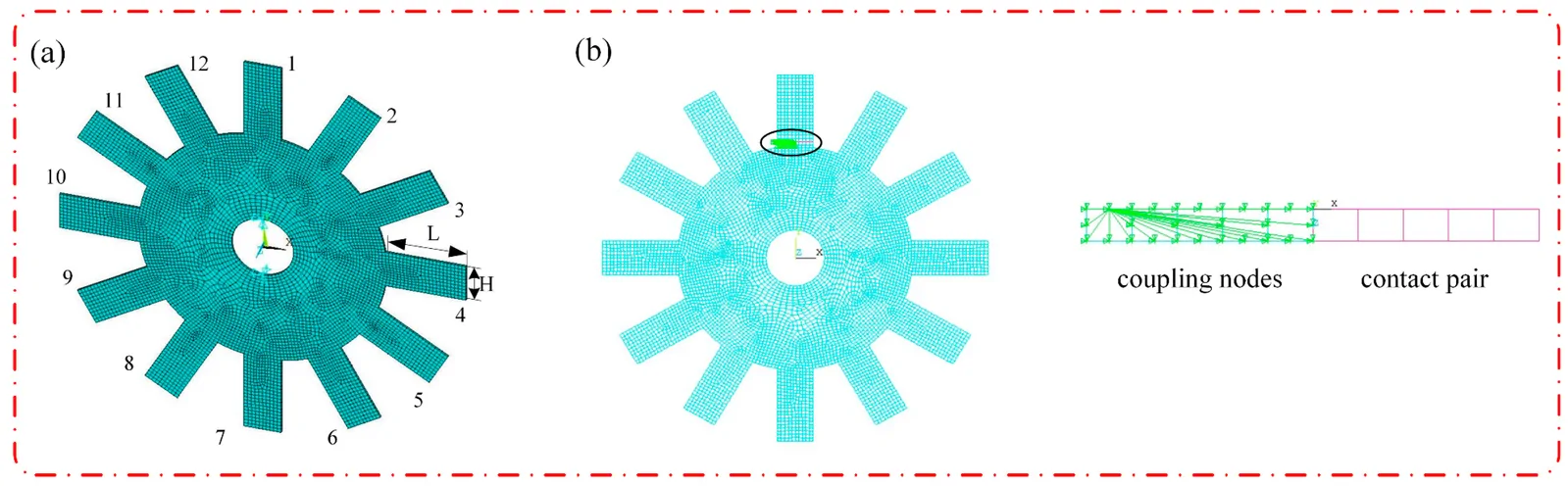
Finite element models: (**a**) the intact blisk and (**b**) the simplified blisk with a breathing crack.

The inner diameter, outer diameter, width, and thickness of the blisk are 50 mm, 200 mm, 30 mm, and 2 mm, respectively, as shown in Figure 5a. In this simplified model, the X− and Y−axes denote the radial directions, while the Z−axis denotes the axial direction. The blisk specimen was made of structural steel (E = 200 GPa, ρ = 7800 kg/m^3^, ν = 0.3). The SOLID186 element was selected, and a swept hexahedral mesh with a 3 mm element size was employed, producing 6433 elements and 46,933 nodes. Fixed constraints were imposed on the inner bore surface nodes, and the natural frequencies and mode shapes were obtained at zero speed. The simplified FE model and boundary conditions are presented in Figure 5a, where the blades are numbered (L: length, H: width). The modeling of the breathing crack in the cracked blisk is as follows. In the uncracked regions of the interface, the two parts are “bonded” together by coupling the degrees of freedom of the coincident nodes. The crack length can be varied by adjusting the number of coupled nodes and the number of contact pairs. In the cracked region, contact pairs are established to simulate the breathing effect of the crack. For these contact pairs, the contact surfaces on the split blade are defined as the contact side and discretized with Conta174 elements, while the corresponding surfaces on the disk body are defined as the target side and discretized with Targe170 elements, as shown in Figure 5b. The cracked interface is modeled using the augmented Lagrangian method for contact, while the uncracked portion is constrained by tying the nodal degrees of freedom. The contact parameters, including factor for the normal stiffness factor (FKN = 1.0), factor for tolerance of normal contact (FTOLN = 0.1), and friction coefficient (μ = 0.3), are defined accordingly. Detailed parameters are listed in Table 1.

The equation of undamped free vibration is expressed as:

(1) $$M\left\{\ddot{u}\left(t\right)\right\}+K\left\{u\left(t\right)\right\}=0$$

where Μ and K are the structural mass matrix and stiffness matrix of the blisk, respectively, and $\left\{u\left(t\right)\right\}$ denotes the displacement vector and represents the acceleration vector.

To extract the natural frequencies and mode shapes, the governing eigenvalue equation for the finite element modal analysis is formulated as:

(2) $$(K-\omega_{i}^{2}M)\Phi_{i}=0$$

where $\omega_{i}^{2}$ and $\Phi_{i}$ denote the i−th natural angular frequency and corresponding mode shape vector of the blisk.

**Figure 6:**
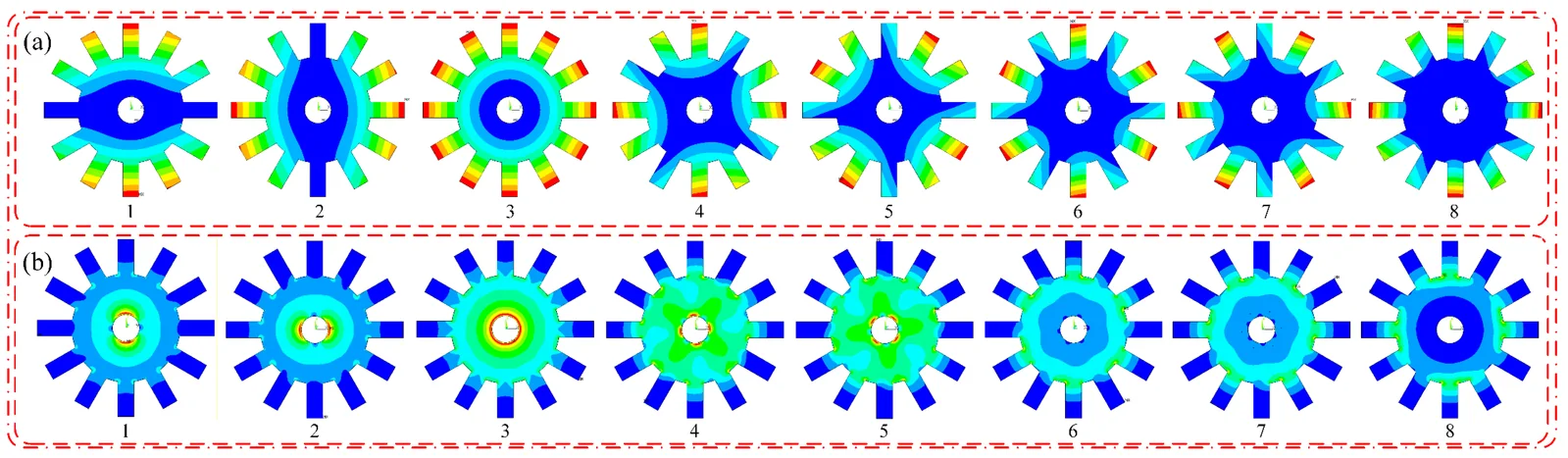
Modal analysis of the simplified blisk: (**a**) first eight mode shapes and (**b**) von Mises stress.

Modal analysis is performed on the simplified blisk under the zero−speed condition, and the resulting first eight mode shapes and von Mises equivalent stress distributions are presented in Figure 6a and Figure 6b, respectively.

**Figure 7:**
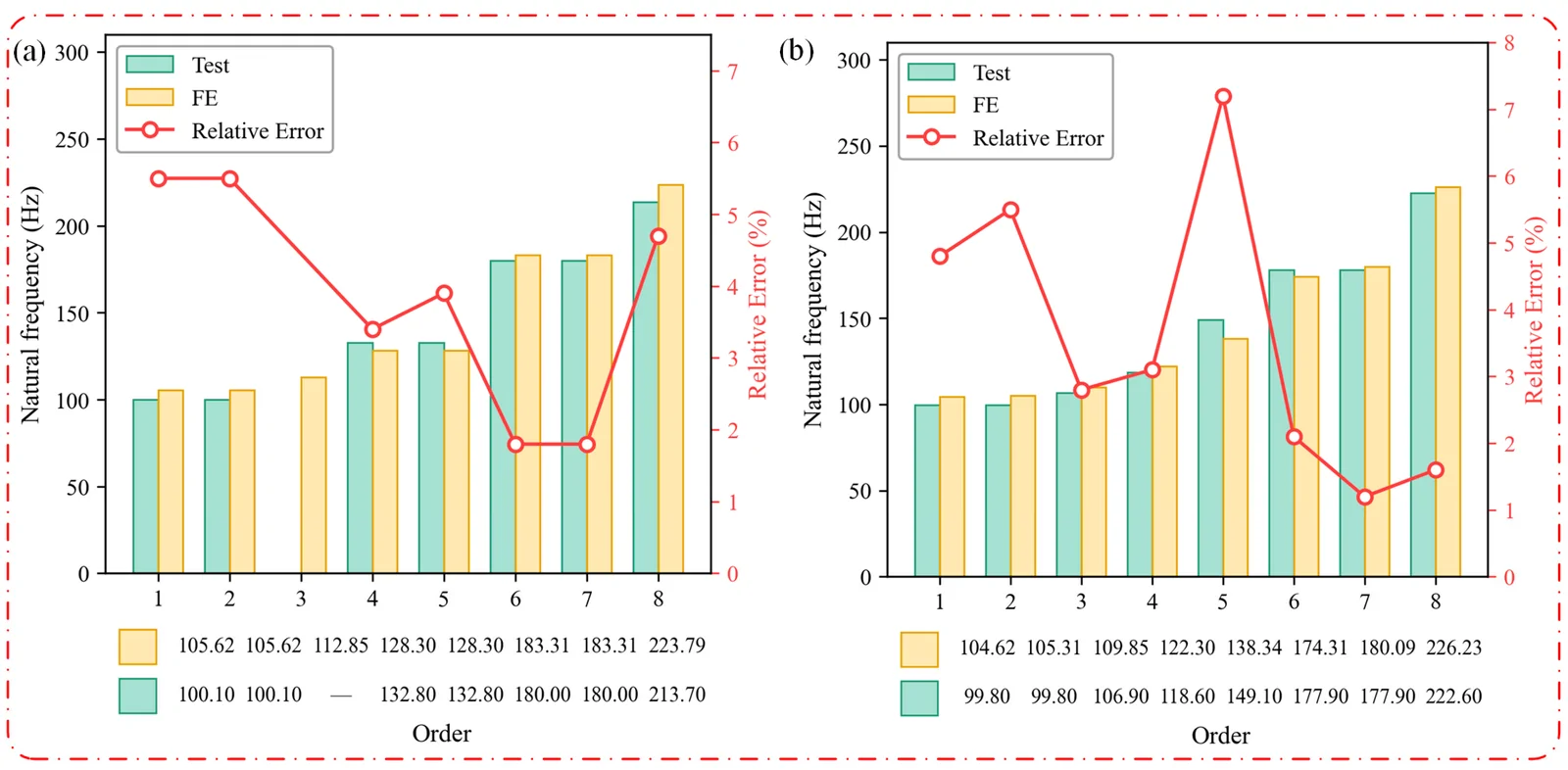
Comparison between experimental and finite element results: (**a**) the intact blisk and (**b**) the blisk with an opening crack.

Comparison of experimental and simulated natural frequencies for intact and cracked blisks shows errors within the 10% engineering tolerance, and the consistent trends confirm the accuracy and validity of the proposed analysis models, as shown in Figure 7.

## 3. Modal Localization Analysis of a Blisk with Breathing Cracks

### 3.1. Modal Analysis of the Intact Blisk and the Blisk with Breathing Cracks

**Figure 8:**
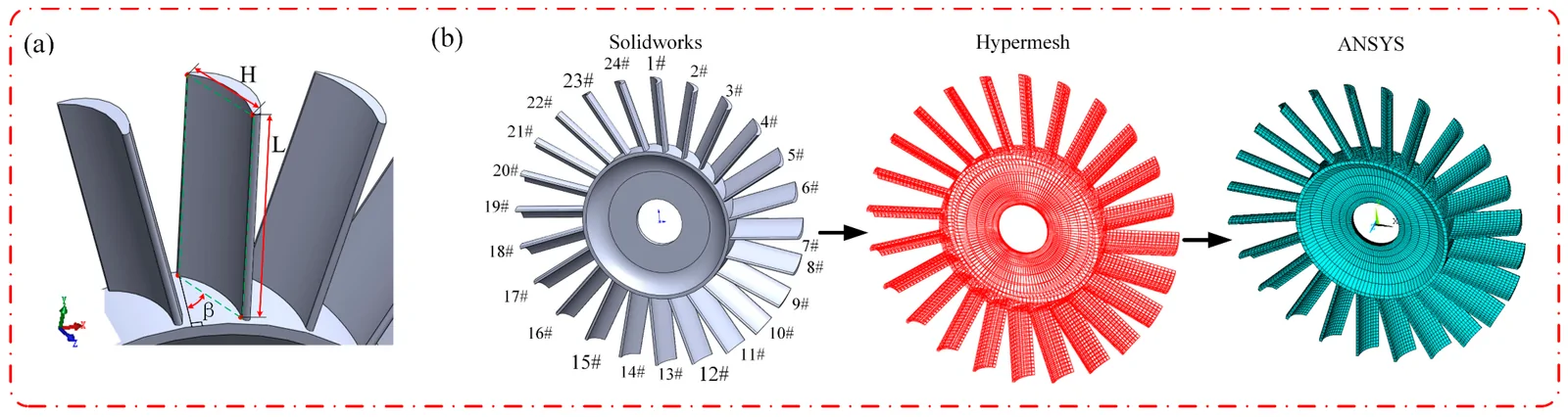
Finite element model of the blisk: (**a**) main geometric parameters and (**b**) modeling procedure.

A tuned blisk represents a completely ideal structure, in which all blades share identical physical properties. However, once breathing cracks appear, the monolithic blisk becomes mistuned, potentially leading to vibration localization. Consequently, the physical properties of each blade sector may differ significantly from one another. Therefore, the model of the integral blisk is employed for analysis, with its main structural parameters and schematic diagram given in Table 2 and Figure 8a, respectively. The modeling process for a tuned integral blisk model is illustrated in Figure 8b, where labels 1# to 24# denote the blade numbers. In this study, TC4 titanium alloy is used for the blisk, with an elastic modulus of 110 GPa, a Poisson ratio of 0.34, and a density of 4440 kg/m^3^. The complex geometry of the blisk was meshed using HyperMesh with 8−node Solid185 hexahedral elements, producing 21,864 elements and 30,816 nodes. A fixed support was applied to the inner ring of the blisk. The two parts are “bonded” together by coupling node degrees of freedom at the non−crack part of the model, and the crack length can be changed by changing the number of nodes of the coupling degree of freedom. As defined in Table 2 and Figure 8a, L and H represent the blade length and width, respectively. The notation 0.9 L × 0.9 H denotes a breathing crack located at a distance of 0.9 L from the blade tip (near the blade root) with a chordwise depth of 0.9 H. The model of a 0.9 L × 0.9 H breathing crack on the 15# blade of the blisk is shown in Section 4.1.

**Figure 9:**
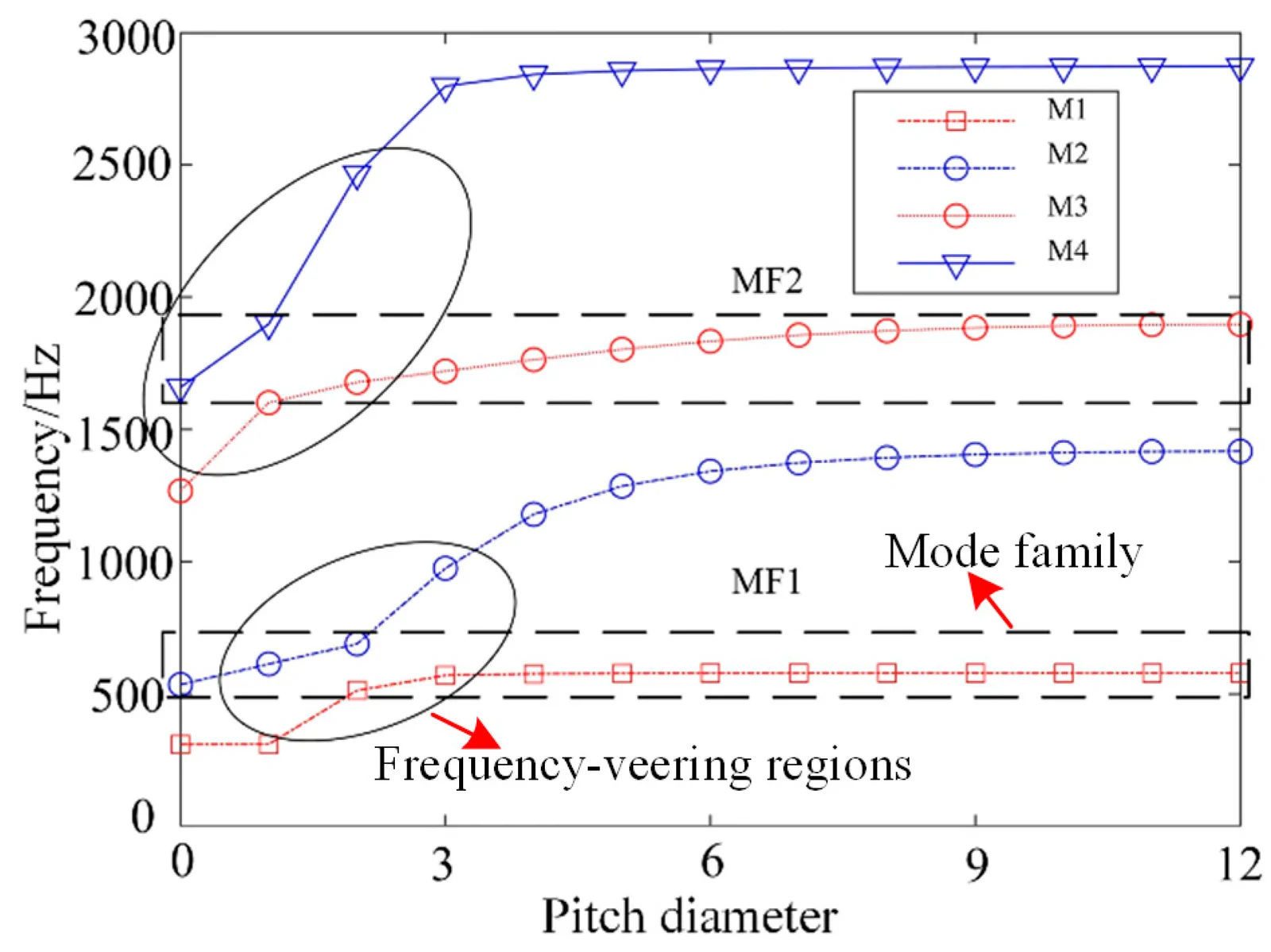
Pitch diameter–frequency diagram of the blisk with modal branches M1–M4 and frequency−veering regions.

The modes of the blisk are mainly divided into the dominant mode of the disk vibration and the dominant mode of blade vibration, and the location of energy convergence is the main basis for their division. The set of all pitch diameters with the same vibration form is called the mode family (MF), and the pitch diameter–frequency curve is an important method to divide the mode families. The prestress modal analysis of the blisk is carried out by ANSYS, and the natural frequency is extracted by the Subspace mode extraction method. The pitch diameter–frequency diagram for the first two modal families of the blisk, with pitch diameter as the horizontal axis and natural frequency as the vertical axis, is shown in Figure 9.

**Figure 10:**
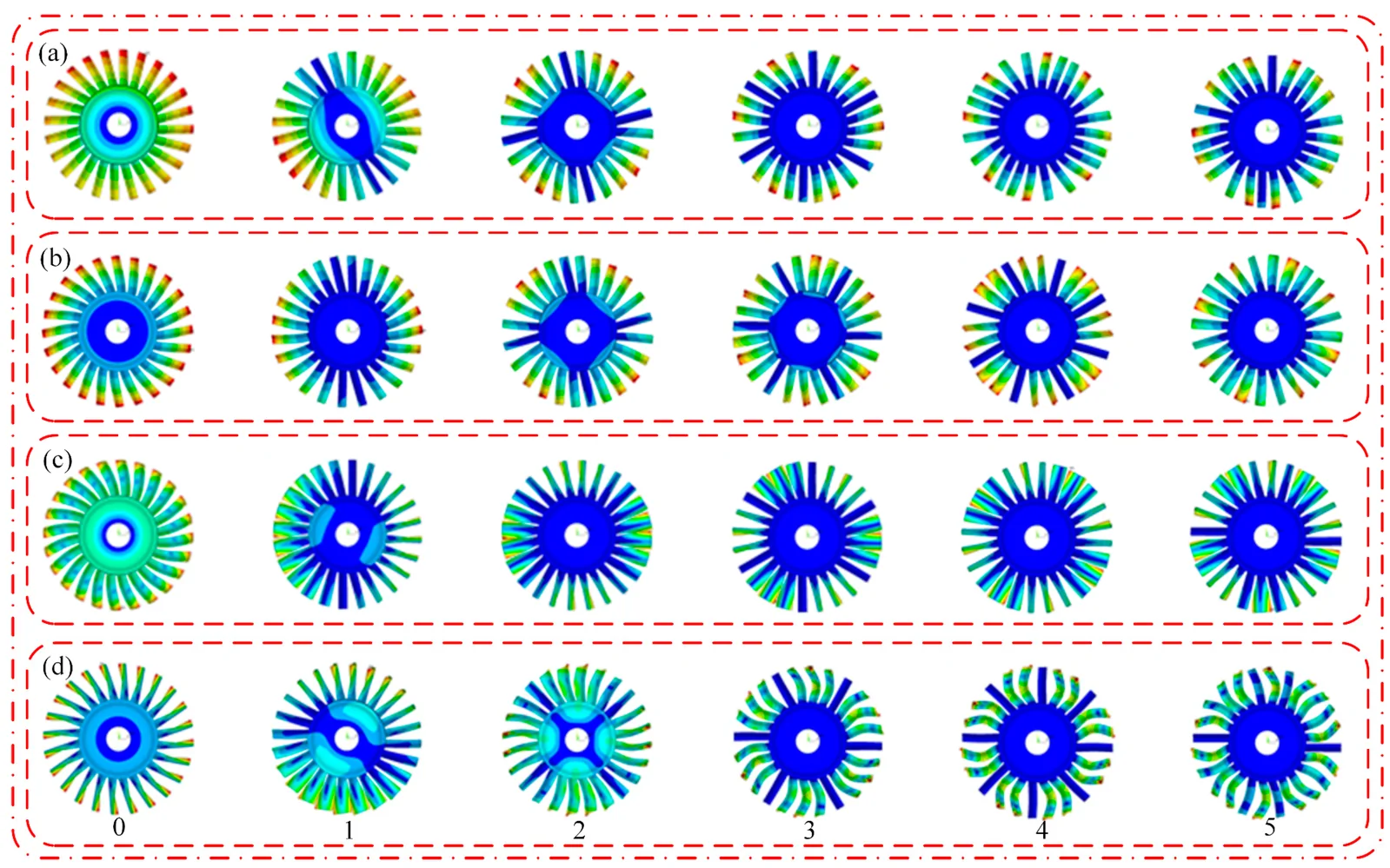
Mode shapes with 0–5 pitch diameters for modal branches: (**a**) M1, (**b**) M2, (**c**) M3, and (**d**) M4.

Through analysis, the following observations are made: For curve M1, the dominant vibration modes of the disk in the integral blisk are those with 0–1 pitch diameters. The blade–disk coupled vibration of the integral blisk occurs in the frequency−veering region for modes with 2–3 pitch diameters. Beyond 3 pitch diameters, the coupled vibration gradually transitions into modes dominated by blade vibration. As this transition occurs, the vibration energy of the blisk gradually shifts from the disk to the blades, and blade bending modes appear after 3 pitch diameters. For curve M2, the dominant blade vibration modes correspond to 0–1 pitch diameter, representing the first bending vibration of the blisk blades. Combined with curve M2, the 2–12 pitch diameter modes of curve M1 form the first bending vibration mode family of the blisk blades. For curve M4, the torsional vibration modes of the blisk blades are identified. Specifically, the mode at 0 pitch diameter represents the torsional vibration of the blisk blades. The 1–12 pitch diameter modes of curve M3 collectively form the torsional mode family of the blisk blades. Comparison between the disk and the blades of the integral blisk reveals that the blades have lower stiffness. Therefore, when the pitch diameter number is large, most vibration modes are dominated by blade vibrations. In the pitch diameter–frequency diagram, the natural frequencies associated with disk−dominated vibrations of the blisk show significant variation, whereas the frequencies of blade−dominated vibrations are densely distributed and approximately form a straight line (Figure 10).

**Figure 11:**
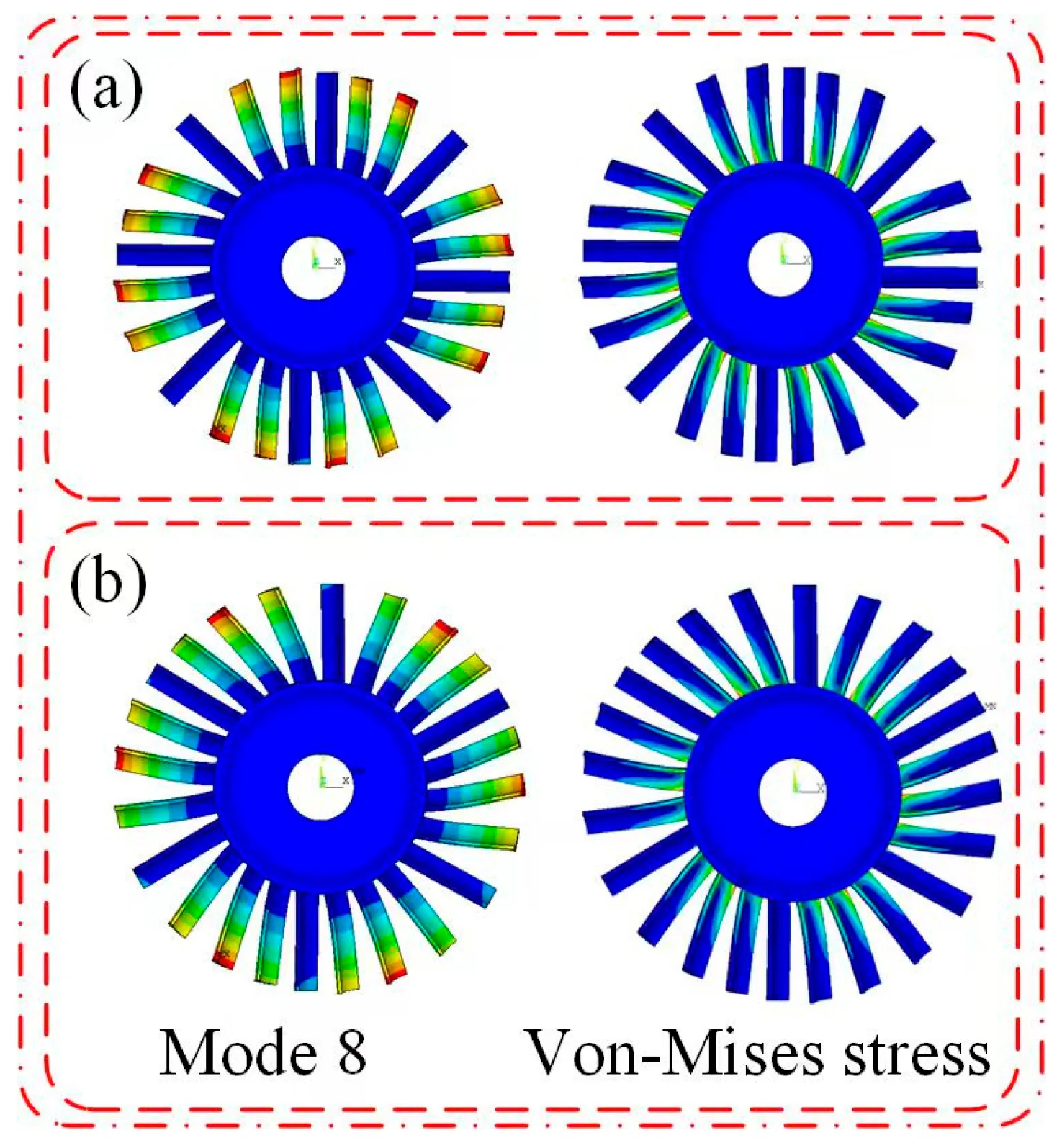
Blisk modal analysis: (**a**) 0 rpm and (**b**) 15,000 rpm.

**Figure 12:**
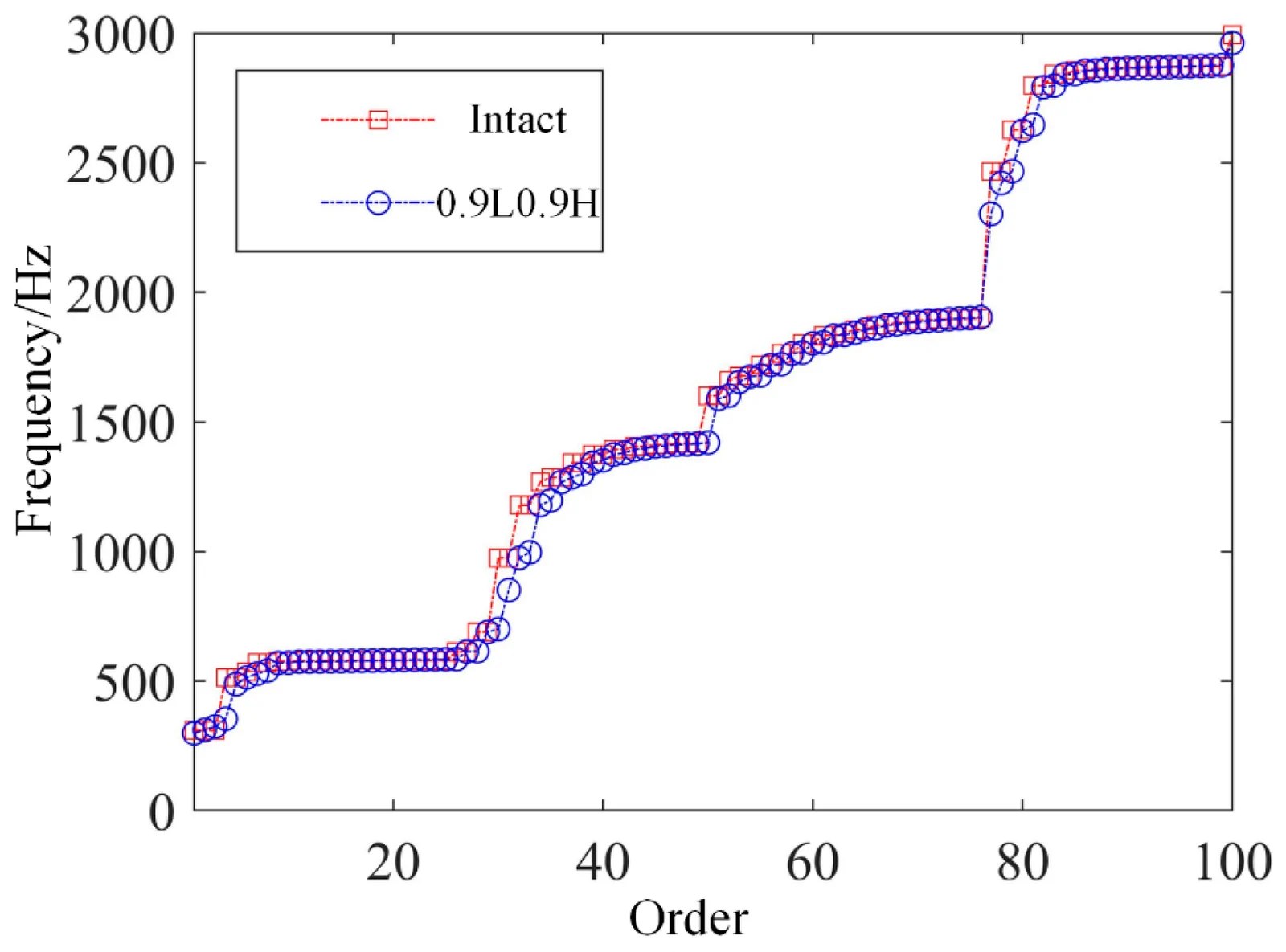
Natural frequencies of intact and cracked blisks.

Through analysis of the mode shapes and von Mises stress distributions of the blisk under non−rotating and rotating (15,000 rpm) conditions, as shown in Figure 11, the eighth−order vibration of blade 15# was found to be particularly pronounced. The maximum stress was concentrated on the blade, and the crack−prone region was located near the blade root. Therefore, the eighth order was selected as the focus order. Subsequently, a breathing crack was introduced at a distance of 0.9 L from the blade tip, with a crack length of 0.9 H. Modal analysis using ANSYS was then performed to obtain the first 100 natural frequencies of the blisk both without and with this breathing crack, and the results are presented in Figure 12. Figure 12 reveals that the periodic symmetry of the blisk is disrupted by the crack, leading to frequency separation. Compared with the intact blisk, the natural frequencies of the cracked blisk show little overall change. Modes dominated by blade vibration still exhibit frequency density, while those dominated by wheel body vibration retain their frequency slope. In some modes, mode localization is observed and is relatively pronounced.

**Figure 13:**
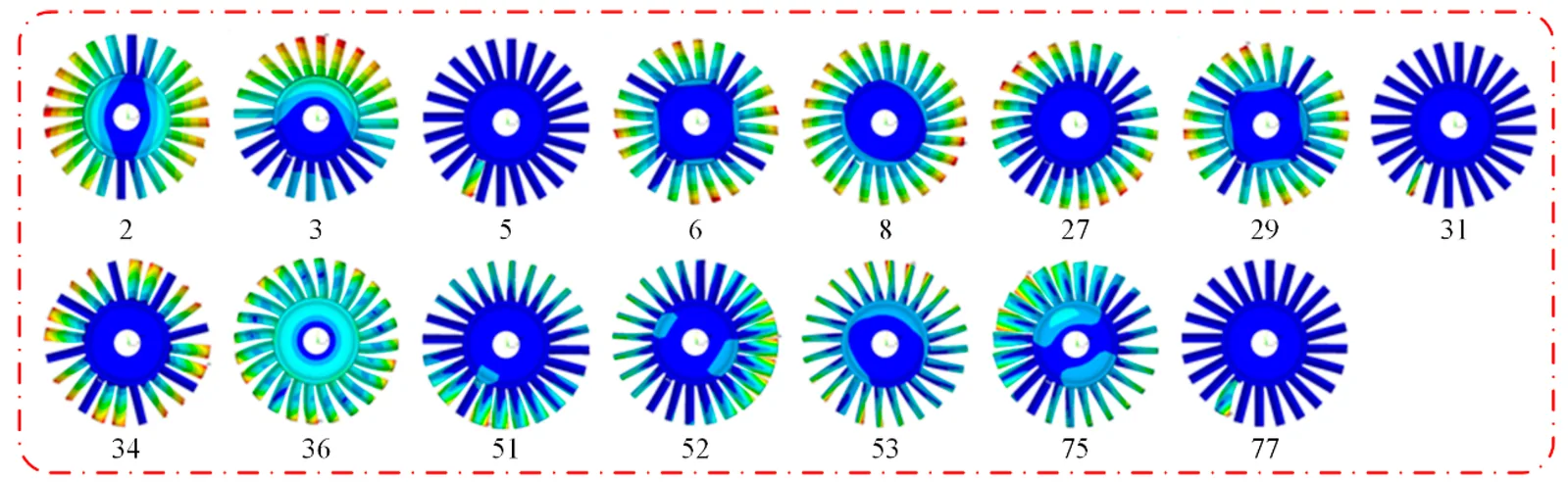
Representative mode shapes of the cracked blisk showing localized veering modes and disk modes.

Figure 13 presents the mode shapes of selected orders for the blisk with a 0.9 L and 0.9 H breathing crack. Analysis reveals that the vibration modes dominated by the disk body of the integral blisk are orders 2, 3, and 36. Among these, the diametral modes (diameter 1 and diameter 0) observed before cracking correspond to orders 2 and 36, respectively. The vibration modes of the intact blisk are also shown in Figure 10, and cracks have little effect on these mode shapes. The modes located in the frequency−veering region include orders 3, 5, and 31, which exhibit coupled disk–blade vibration; these modes become localized after crack initiation. The remaining modes are dominated by blade vibration, and significant mode localization occurs after cracking. Therefore, the effect of breathing cracks on the modal characteristics of the integral blisk can be summarized as follows. Breathing cracks are highly sensitive to modes in the frequency−veering region and to mode groups dominated by blade vibration, but they have almost no influence on modes dominated by the disk body of the integral blisk. For clear modal classification and verifiable parameter mapping, Table 3 systematically summarizes these representative modes, including their nodal diameters, affiliated frequency branches, and information on whether each mode falls within the frequency−veering region.

### 3.2. The Influence of Crack Distribution on the Inherent Characteristics of a Blisk with Breathing Cracks

The localization factor is an important way to quantitatively describe the degree of localization of the blisk with breathing cracks. Since the mode is a dimensionless relative vibration quantity, the corresponding modal displacement of each blade in the modal mode represents the ratio of vibration energy of each blade in the total energy [30]. Therefore, the mode localization factor of a blisk with breathing cracks can be defined as:

(3) $$R=\frac{U_{m}-U_{n}}{U_{n}}$$

where $U_{m}$ and $U_{n}$, respectively, represent the maximum dimensionless mode displacement of the integral blisk before and after breathing cracks.

**Figure 14:**
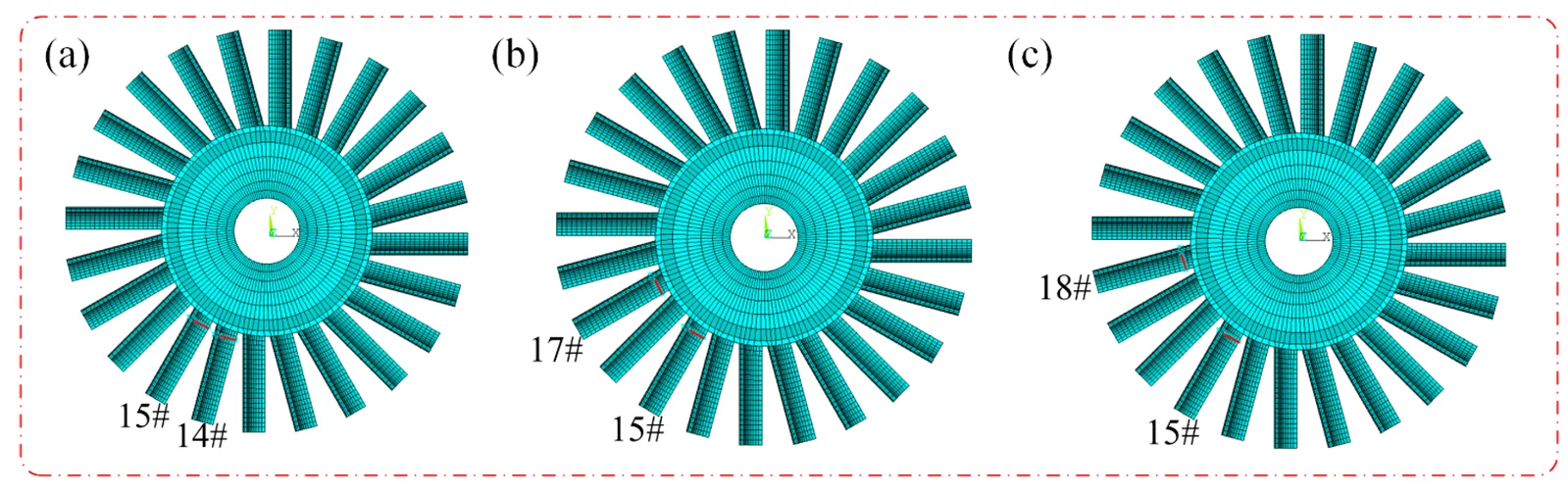
Three integral blisk crack patterns: (**a**) adjacent, (**b**) 1 blade apart, and (**c**) 2–blades apart.

In this section, the effect of the distribution of blades with two breathing cracks on the vibration characteristics of the blisk is studied, which provides ideas and methods for analyzing the situation of the blisk with multiple breathing cracks. It is known that the breathing crack is very sensitive to the mode family of the blisk and the mode of the frequency−veering region, so the degree of localization of these modes is focused on. The main research object in this section is the integral blisk with adjacent cracks (adjacent), separated by 1 complete blade crack (separated by 1), separated by 2 complete blade cracks (separated by 2), and the integral blisk models with three different crack distributions, as shown in Figure 14.

The finite element model of the blisk with different crack distributions is numerically simulated. Sensitive modes of both the intact blisk and the blisk with breathing cracks are extracted near the mode families and the frequency−veering regions. Specifically, Modes 3 and 5 are selected as representative cases because they lie within frequency−veering regions (at the 2nd and 3rd nodal diameters on the M1 branch) and exhibit the highest modal density, thereby forming the strongest disk–blade coupling effect that makes the system highly sensitive to crack−induced mistuning perturbations. The tip displacement of each blade is taken as the modal displacement of that blade. By comparing the modal displacements of the entire blisk under different crack distributions, the degree of modal localization can be quantitatively characterized.

**Figure 15:**
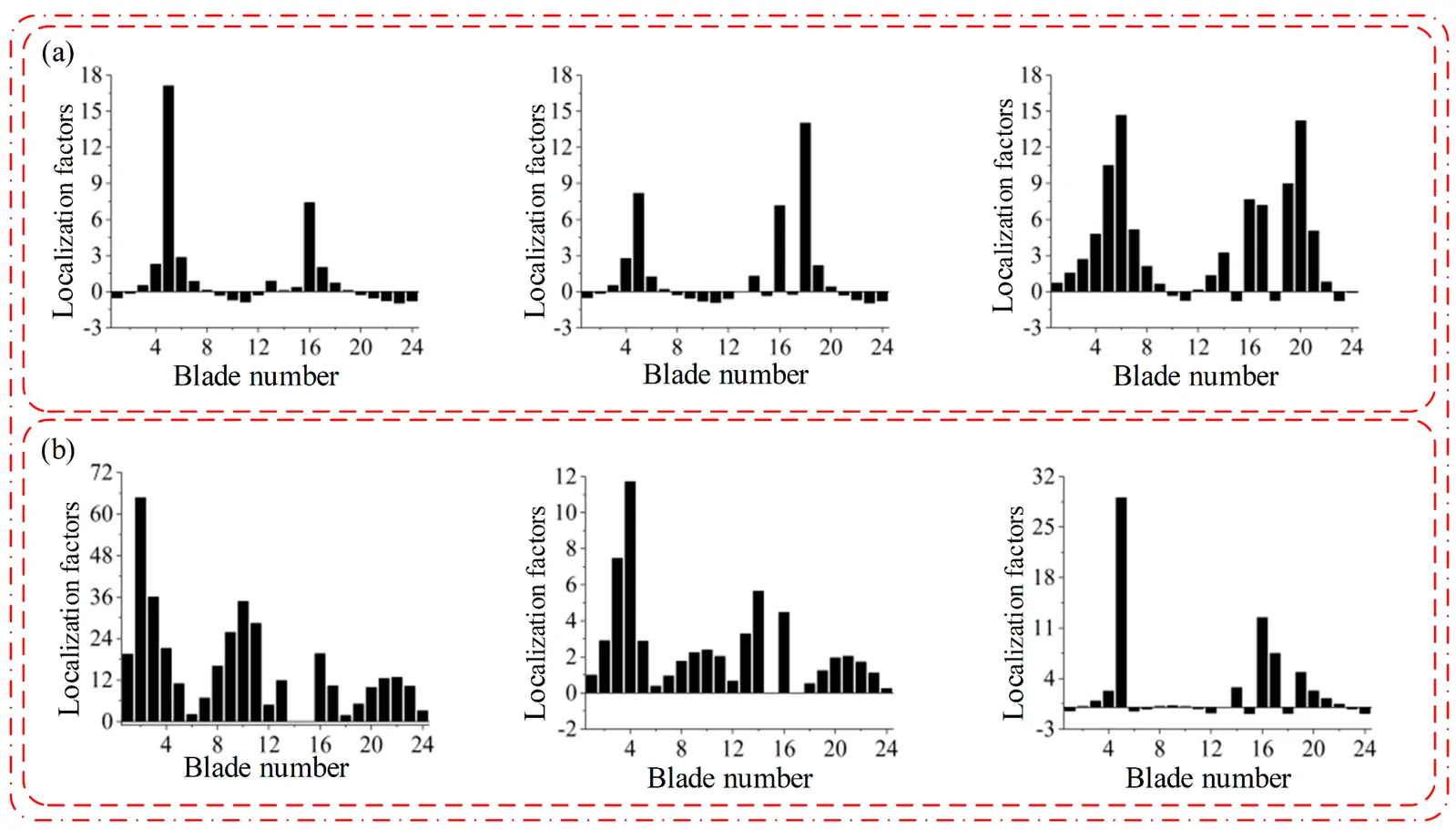
Localization factors for cracks separated by adjacent crack distributions, one blade and two blades: (**a**) Mode 3 and (**b**) Mode 5.

From Figure 15, the following observations can be made: (a) An obvious mode localization phenomenon occurs in the sensitive mode order near the frequency−veering region. Only one or a few blades exhibit a high mode localization factor, whereas the remaining blades show a small or even negative localization factor, indicating that no mode−localized vibration is induced in those blades. (b) Figure 15 reveals that mode localization does not occur on the blades containing breathing cracks in the blisk. When the cracks are adjacently distributed (i.e., on blades 14# and 15#), the largest localization factors appear on blades 5# and 2#, with blades 2# and 14# being symmetrically positioned. This phenomenon is particularly pronounced when the frequency−veering gap is at its minimum. In addition, localized vibration is also evident on the adjacent blade 16#. For crack distributions with one or two intact blades separating the cracked blades, localized vibration is significant on the symmetrically positioned blades, as well as on the intact blades located between the cracked ones. (c) Among the different crack distribution patterns, the adjacent crack distribution results in the highest degree of modal localization in the blisk.

### 3.3. Influence of Blade Twist Angle on the Natural Characteristics of a Blisk with Breathing Cracks

**Figure 16:**
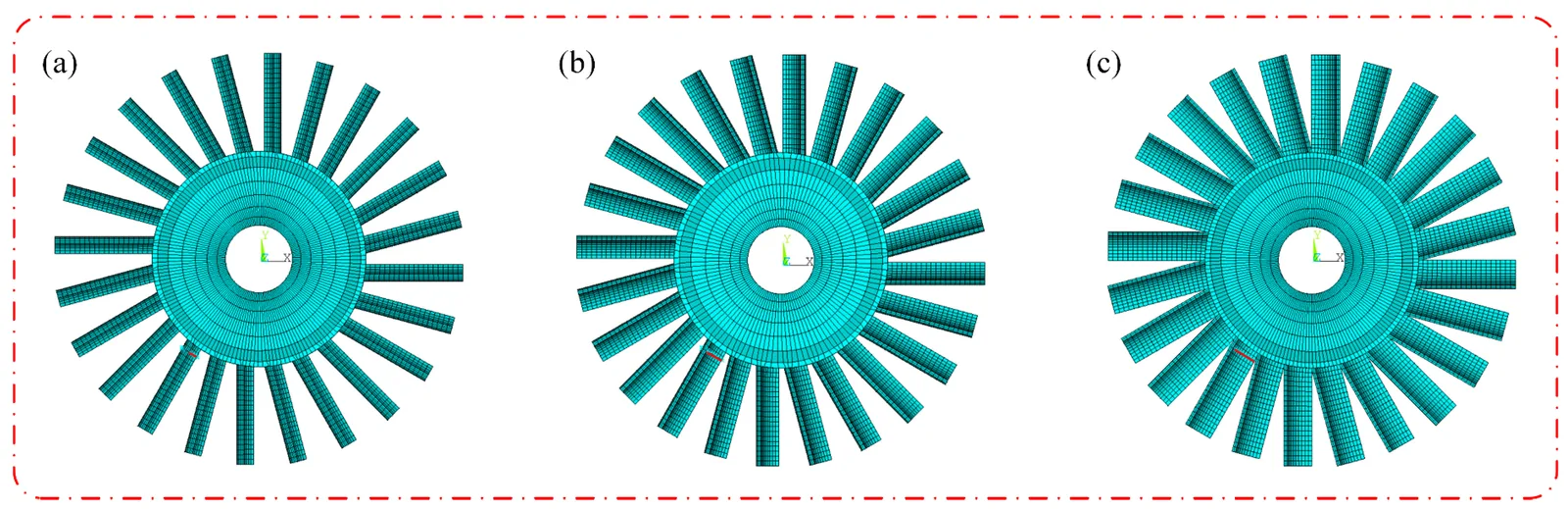
The blisk of three blade angles: (**a**) angle 15°, (**b**) angle 30°, and (**c**) angle 45°.

In this section, integral blisks containing breathing cracks with blade angles of 15°, 30°, and 45° are selected for investigation. In all cases, a 0.9 L × 0.9 H breathing crack is introduced in blade 15#. The finite element models of the blisks with the three different blade angles are shown in Figure 16. Numerical simulations were conducted on blisk models with different blade angles and breathing cracks. For the intact and cracked blisks, the displacements at the blade tips were extracted from the crack−sensitive modes near the frequency−veering region and used as the blade modal displacements. The modal displacement variations in the integral blisk at different blade angles were then compared for selected modes, thereby enabling a quantitative evaluation of the degree of modal localization induced by breathing cracks under different blade−angle conditions.

**Figure 17:**
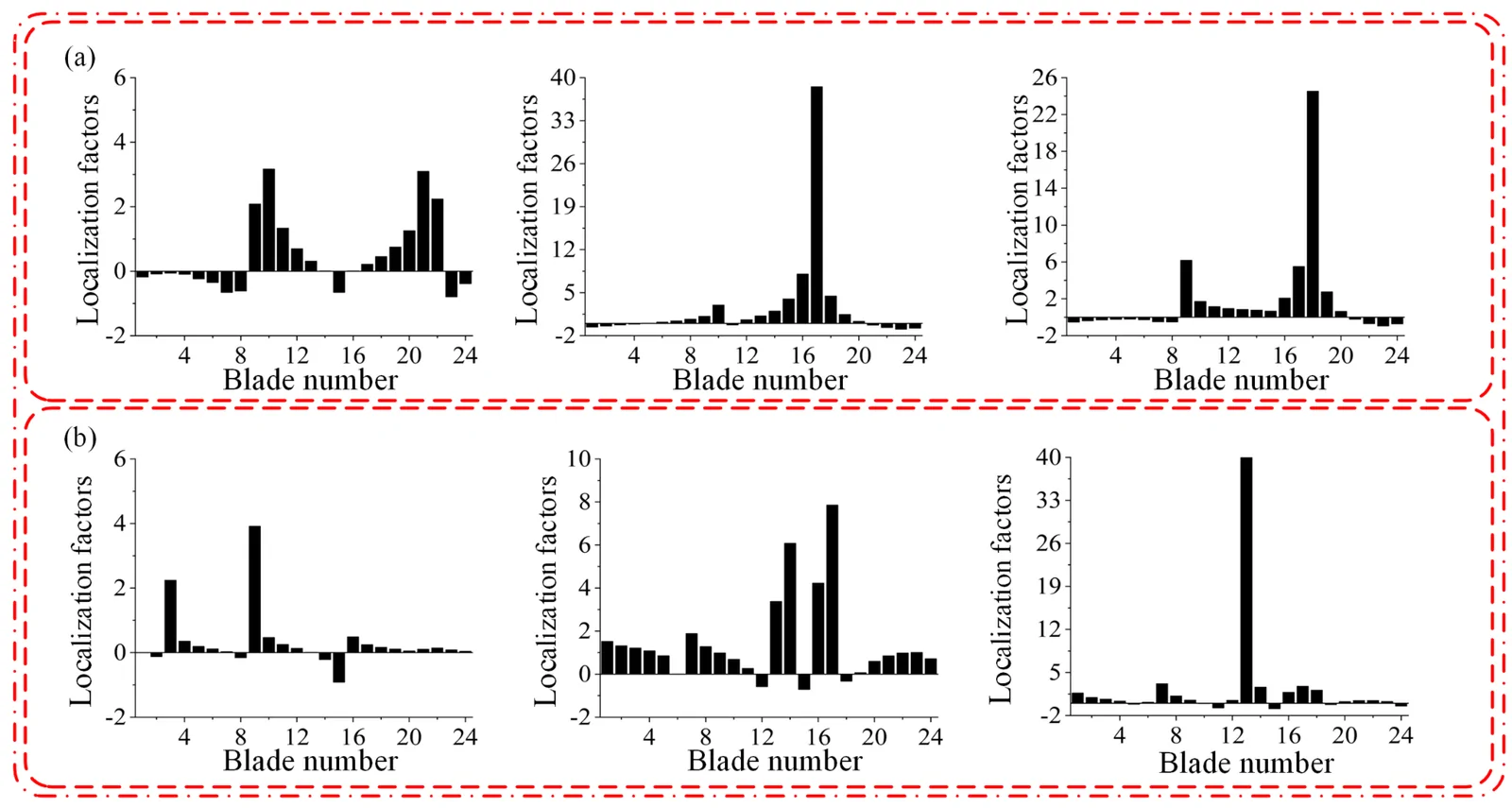
Localization factor of angles 15°, 30°and 45°: (**a**) Mode 3 and (**b**) Mode 5.

As shown in Figure 17, the following observations can be made. (a) When the blade angle is 15°, the modal localization phenomenon is relatively weak. As the blade angle increases to 30° and 45°, the localization factor increases, indicating a higher degree of modal localization. (b) In the case of a single crack, the blade with the maximum localization factor does not appear at a symmetric position, which is markedly different from the pattern observed in the integral blisk with two breathing cracks. Moreover, the most pronounced modal localization does not occur on the cracked blade itself, but rather on the adjacent blade. (c) Significant modal localization is observed either in one or a few blades.

## 4. Transient Response Analysis of a Blisk with Breathing Cracks

### 4.1. Transient Analysis of the Intact and Cracked Blisk

The full transient dynamic method was employed to analyze the response characteristics of the blisk.

(4) $$M\left\{\ddot{u}\left(t\right)\right\}+C\left\{\dot{u}\left(t\right)\right\}+K\left\{u\left(t\right)\right\}=\left\{F\left(t\right)\right\}$$

where M, C and K are the mass, damping, and stiffness matrices of the blisk, respectively; $\left\{F\left(t\right)\right\}$ is the excitation force vector; $\left\{\ddot{u}\left(t\right)\right\},\;\left\{\dot{u}\left(t\right)\right\},\;\mathrm{and}\;\left\{u\left(t\right)\right\}$ denote the acceleration, velocity, and displacement vectors, respectively; and t is the duration of applied loading.

The Newmark method is a widely used algorithm for transient analysis, which discretizes the time domain via finite differences. Within each time interval, the following recurrence relations can be given:

(5) $$\left\{{\dot{u}}_{n+1}\right\}=\left\{{\dot{u}}_{n}\right\}+\left[\left(1-\delta \right)\left\{{\dot{u}}_{n}\right\}+\delta \left\{{\ddot{u}}_{n+1}\right\}\right]\Delta t$$

(6) $$\left\{u_{n+1}\right\}=\left\{u_{n}\right\}+\left\{{\dot{u}}_{n}\right\}\Delta t+\left[\left(\frac{1}{2}-\alpha \right)\left\{{\ddot{u}}_{n}\right\}+\alpha \left\{{\ddot{u}}_{n+1}\right\}\right]\Delta t^{2}$$

where $\Delta {t=t}_{n}-t_{n-1}$ and α,δ are the Newmark integration parameters (ANSYS default: α=0.25,δ=0.5).

The primary objective of the transient analysis is to solve for $\left\{u_{n+1}\right\}$ at the next time step. Thus, the governing equation at time $t_{n+1}$ is:

(7) $$M\left\{{\ddot{u}}_{n+1}\right\}+C\left\{{\dot{u}}_{n+1}\right\}+K\left\{u_{n+1}\right\}=\left\{F_{n+1}\right\}$$

Expressing acceleration and velocity in terms of displacement yields:

(8) $$\left\{{\ddot{u}}_{n+1}\right\}=\frac{1}{\alpha \Delta t^{2}}\left(\left\{u_{n+1}\right\}-\left\{u_{n}\right\}\right)-\frac{1}{\alpha \Delta t}\left\{{\dot{u}}_{n}\right\}-\left(\frac{1}{2\alpha }-1\right)\left\{{\ddot{u}}_{n}\right\}$$

(9) $$\left\{{\dot{u}}_{n+1}\right\}=\frac{\delta }{\alpha \Delta t}\left(\left\{u_{n+1}\right\}+\left\{u_{n}\right\}\right)+\left(1-\frac{\delta }{\alpha }\right)\left\{{\dot{u}}_{n}\right\}+\Delta t\left(1-\frac{\delta }{2\alpha }\right)\left\{{\ddot{u}}_{n}\right\}$$

Substituting into the equation of motion at time $t_{n+1}$ and combining these equations yields the effective stiffness equation:

(10) $$\hat{K}\left\{u_{n+1}\right\}=\left\{{\hat{F}}_{n+1}\right\}$$

where the effective stiffness matrix $\hat{K}$ is defined as:

(11) $$\hat{K}=K+\frac{1}{\alpha \Delta t^{2}}M+\frac{\delta }{\alpha \Delta t}C$$

The expanded expression for the effective load vector $\left\{{\hat{F}}_{n+1}\right\}$ is given by:

(12) $$\{{\hat{F}}_{n+1}\}=\{F_{n+1}\}+M\left[\frac{1}{\alpha \Delta t^{2}}\{u_{n}\}+\frac{1}{\alpha \Delta t}\{{\dot{u}}_{n}\}+\left(\frac{1}{2\alpha }-1\right)\{{\ddot{u}}_{n}\}\right]+C\left[\frac{\delta }{\alpha \Delta t}\{u_{n}\}+\left(\frac{\delta }{\alpha }-1\right)\{{\dot{u}}_{n}\}+\frac{\Delta t}{2}\left(\frac{\delta }{\alpha }-2\right)\{{\ddot{u}}_{n}\}\right]$$

After solving for $\left\{u_{n+1}\right\}$, back−substitute to calculate $\left\{{\dot{u}}_{n+1}\right\}$ and $\left\{{\ddot{u}}_{n+1}\right\}$, and proceed to the next time step.

Rayleigh damping was employed to compute the mass−proportional α and stiffness−proportional β damping coefficients for the blisk transient response:

(13) C=αM+βK

where α is the mass damping coefficient, M is the mass matrix, β is the stiffness damping coefficient, and K is the stiffness matrix.

According to the principle of orthogonality, the damping ratio $\xi_{r}$ and natural frequency of any order mode satisfy:

(14) $$\xi_{r}=\frac{1}{2}\left(\frac{\alpha }{\omega_{r}}+\beta \omega_{r}\right)$$

where $\omega_{r}$ is the natural frequency of the *r*th order, r=1,2,3,…,n, $\omega_{i}$ and $\omega_{j}$ are the *i*th and *j*−th order frequencies of the blade, and the corresponding modal damping ratios are $\xi_{i}$ and $\xi_{j}$, respectively, and substituting into Equation (14) yields:

(15) $$\alpha =\frac{2\omega_{i}\omega_{j}\left(\xi_{i}\omega_{j}-\xi_{j}\omega_{i}\right)}{\omega_{j}^{2}-\omega_{i}^{2}};\beta =\frac{2\left(\xi_{j}\omega_{j}-\xi_{i}\omega_{i}\right)}{\omega_{j}^{2}-\omega_{i}^{2}}$$

**Figure 18:**
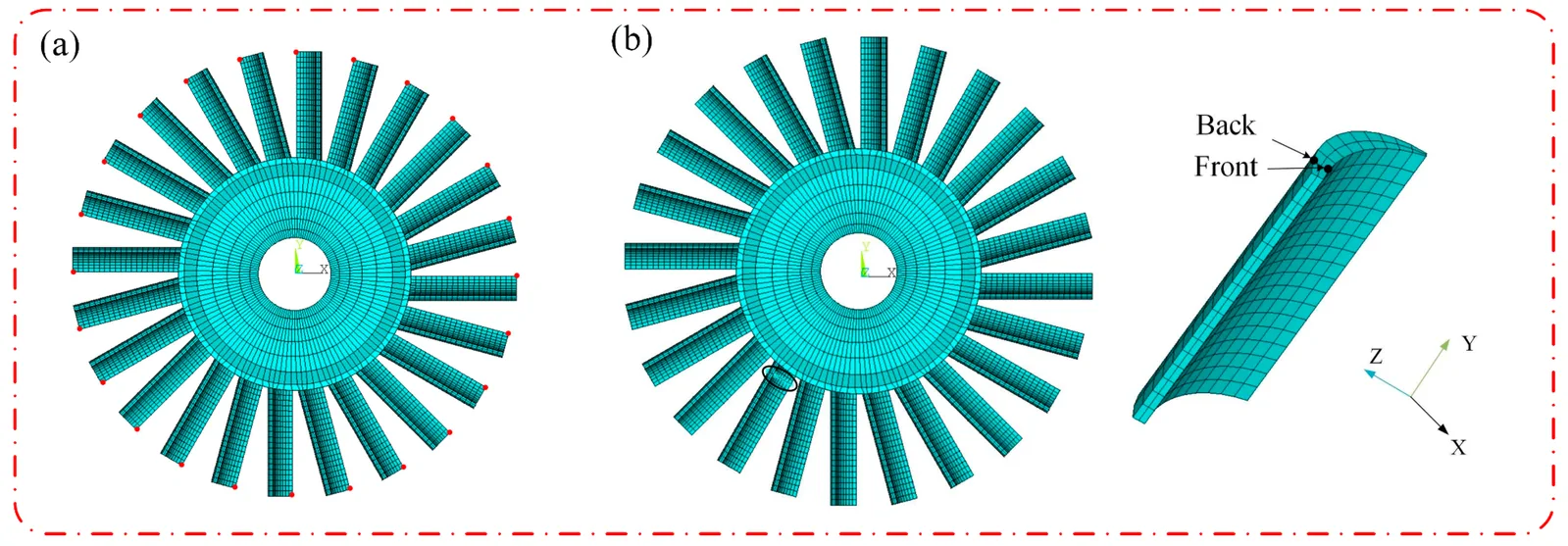
Finite element model of the cracked blisk: (**a**) excitation point and pickup point and (**b**) contact pressure pick point.

To ensure adequate solution accuracy, the time integration step was selected to be less than one−twentieth of the reciprocal of the frequency of interest. Since the blisk was made of TC4 titanium alloy, the Rayleigh damping coefficients were recalculated accordingly, α=3.6033,β=0.00000053957. An axial excitation load of $F=100\times \sin\left(2\pi ft\right)$ was applied at the tip of each blade. The locations of the excitation and response measurement points are shown in Figure 18a. The response characteristics of the blisk are investigated over a rotational speed range of 0–15,000 r/min. In the transient analysis, the large deflection option is activated to account for the rotational stiffening effect, and the crack displacement on the contact pair under centrifugal loading is calculated.

**Figure 19:**
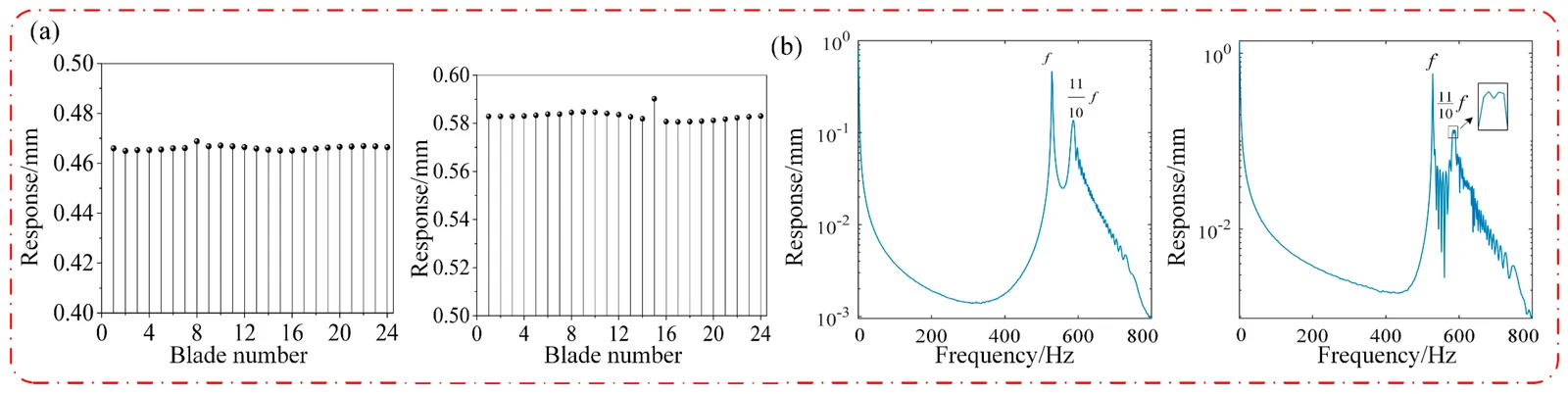
Tip response characteristics of the blisk without cracks and with a 0.9 L × 0.9 H crack: (**a**) Per−blade amplitudes and (**b**) 15# tip response spectrum.

Transient response analysis was performed on the intact and cracked blisk under variable rotational speed conditions, and the vibration amplitudes at the tips of all 24 blades were obtained, as shown in Figure 19. The results indicate that the amplitudes of all blades are nearly identical, demonstrating that the intact blisk maintains cyclic symmetry and periodicity in the absence of cracks. However, when a crack appears at the root of Blade 15#, the blade amplitudes exhibit significant differences, as seen in Figure 19a. The frequency response spectrum of blade 15# of the intact and cracked blisk is shown in Figure 19b. The vibration response of the blisk reaches a peak when the eighth−order natural frequency coincides with the excitation frequency (528.78 Hz), indicating that resonance occurs. In the vibration response spectrum, besides the fundamental frequency, a super−harmonic component at 11/10 times the excitation frequency is also detected, which corresponds to 581.658 Hz and 580.877 Hz for the intact and cracked blisk, respectively. Further observation reveals that the crack causes a reduction in the frequency corresponding to the peak response, generates several small peaks near the super−harmonic component, and significantly increases the response amplitude relative to that of the intact blisk. These results demonstrate that the presence of a breathing crack leads to increasingly pronounced response localization.

**Figure 20:**
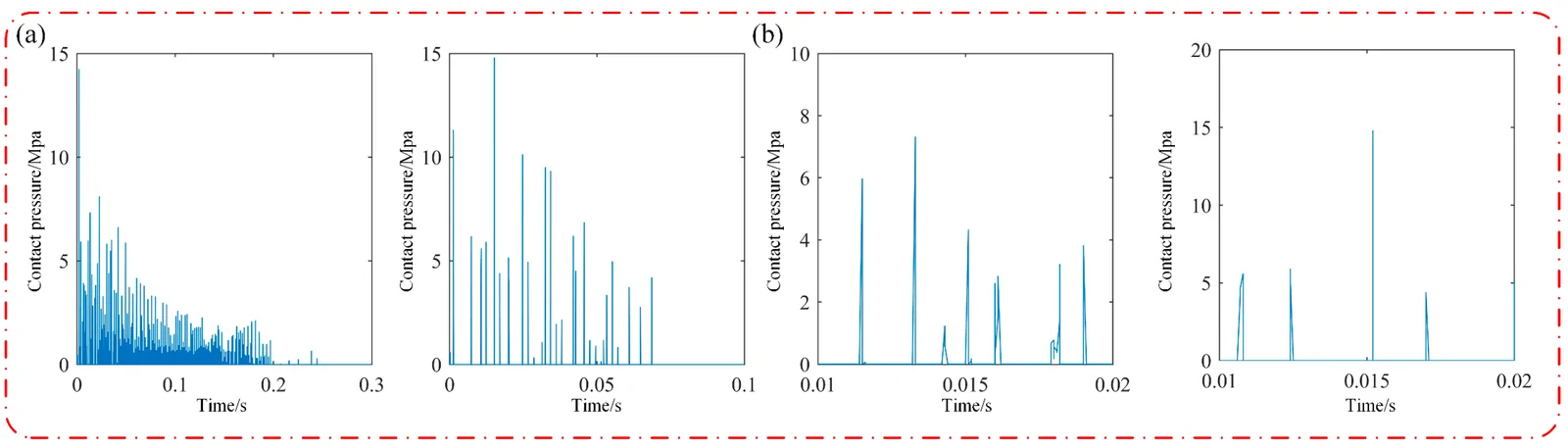
Time–domain response of front point and back point contact pressure: (**a**) contact pressure and (**b**) local magnification.

As shown in Figure 20, the time–domain contact pressure responses at the front and back monitoring points were extracted (see Figure 18b) to analyze the contact pressure characteristics at the contact interface. The results confirm that the crack exhibits a distinct breathing behavior.

### 4.2. Effect of Crack Distribution on the Response Characteristics of a Blisk with Breathing Cracks

**Figure 21:**
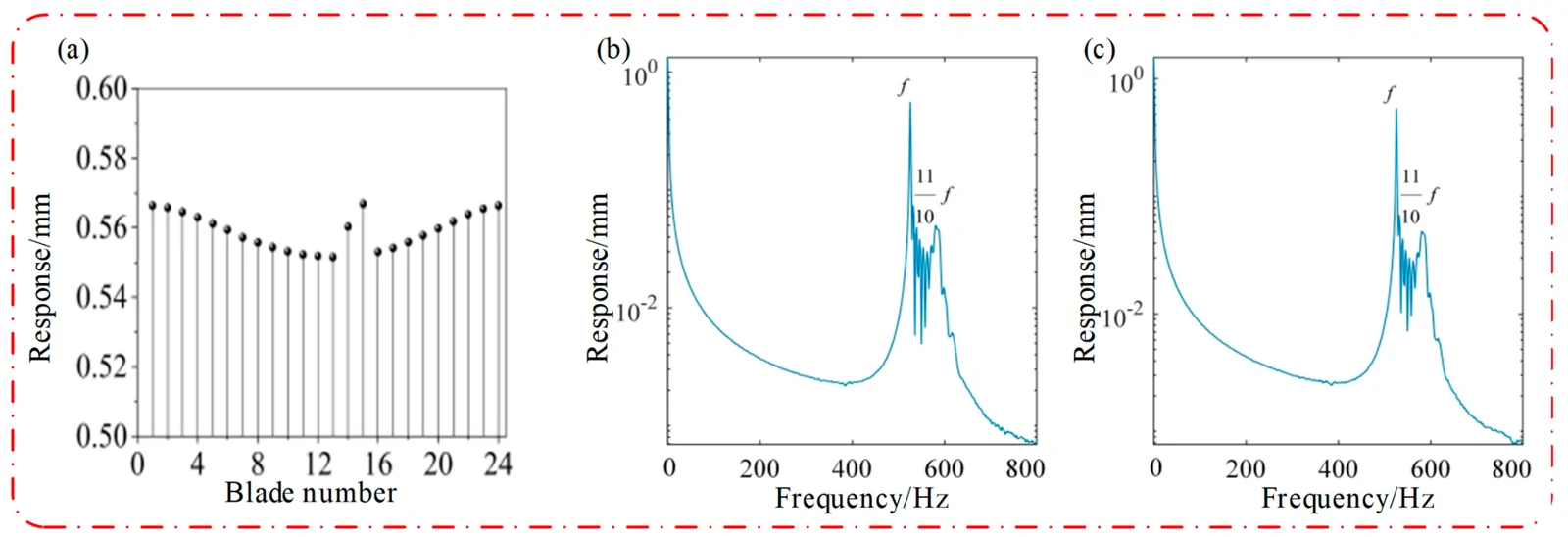
Tip response of the blisk with cracks on adjacent blades: (**a**) Per–blade amplitudes and (**b**) 14#, (**c**) 15#.

**Figure 22:**
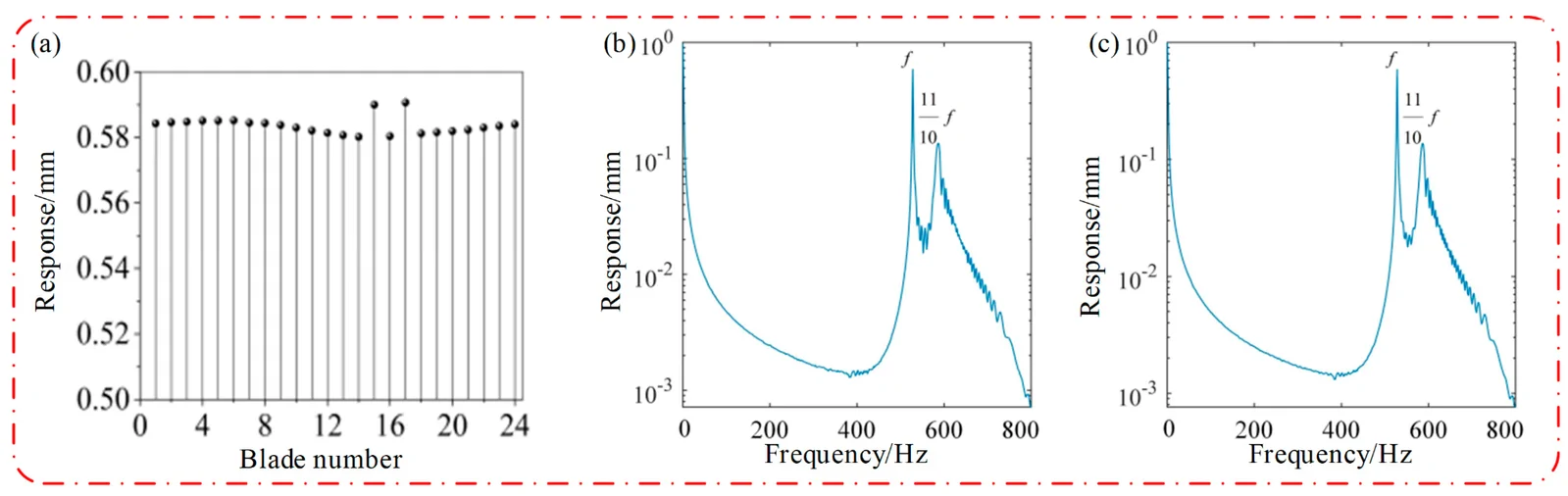
Tip response of the blisk with cracks separated by 1 blade: (**a**) Per–blade amplitudes, (**b**) 15#, and (**c**) 17#.

**Figure 23:**
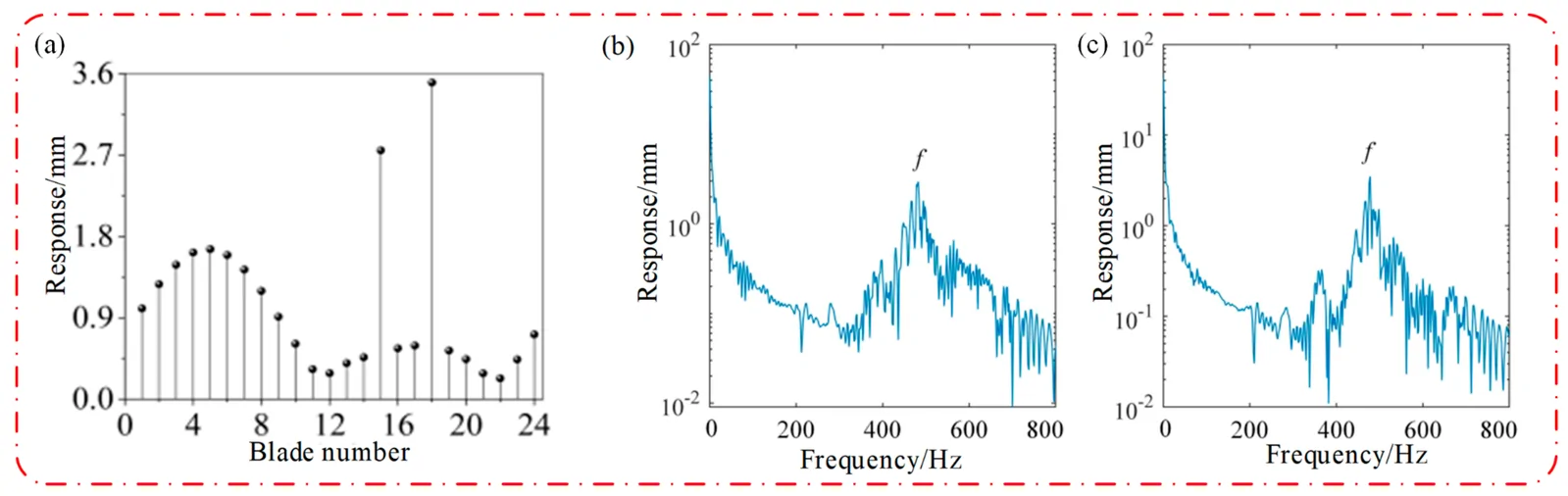
Tip response of the blisk with cracks separated by 2 blades: (**a**) Per–blade amplitudes, (**b**) 15#, and (**c**) 18#.

In this section, the effect of crack distribution on the response characteristics of the blisk is examined. The blade−tip displacement amplitudes are evaluated under variable rotational speed conditions for three crack configurations: adjacent cracks, cracks separated by one intact blade, and cracks separated by two intact blades. The amplitudes of all 24 blades are compared. As illustrated in Figure 21a, Figure 22a and Figure 23a, compared with the single−crack case, the adjacent−crack configuration reduces the blade−tip response amplitudes, exhibiting a vibration suppression effect. In contrast, configurations with one or two intact blades between cracks lead to a significant increase in response amplitudes, with the two−intact−blade case exhibiting the largest amplitudes, exceeding those of the other configurations by more than twice. Notably, the blade containing the breathing crack shows a response amplitude approximately six times that of the remaining blades. The non−uniform circumferential distribution of amplitudes among the other blades indicates that the crack induces significant response localization in the blisk structure.

As shown in Figure 21b,c, Figure 22b,c and Figure 23b,c, the vibration response spectra under different crack distribution patterns exhibit not only the excitation frequency but also pronounced super−harmonic components at approximately 1.1 times the excitation frequency. Specifically, for the adjacent−crack case, a super−harmonic component at 578.31 Hz is observed in addition to the excitation frequency of 525.74 Hz. For the configuration with one intact blade between cracks, the response spectrum contains the excitation frequency (527.92 Hz) along with a corresponding super−harmonic at 580.71 Hz. When the cracks are separated by two intact blades, the dominant excitation frequency shifts to 477.48 Hz. A comparison among these cases reveals that the introduction of breathing cracks causes the peak−response frequency to shift toward lower frequencies, with the most pronounced effect observed in the two−intact−blade separation case, where multiple peaks emerge at the response peak. These features indicate stronger nonlinear behavior and a more localized response.

### 4.3. The Influence of Blade Angle on the Response Characteristics of a Blisk with Breathing Cracks

**Figure 24:**
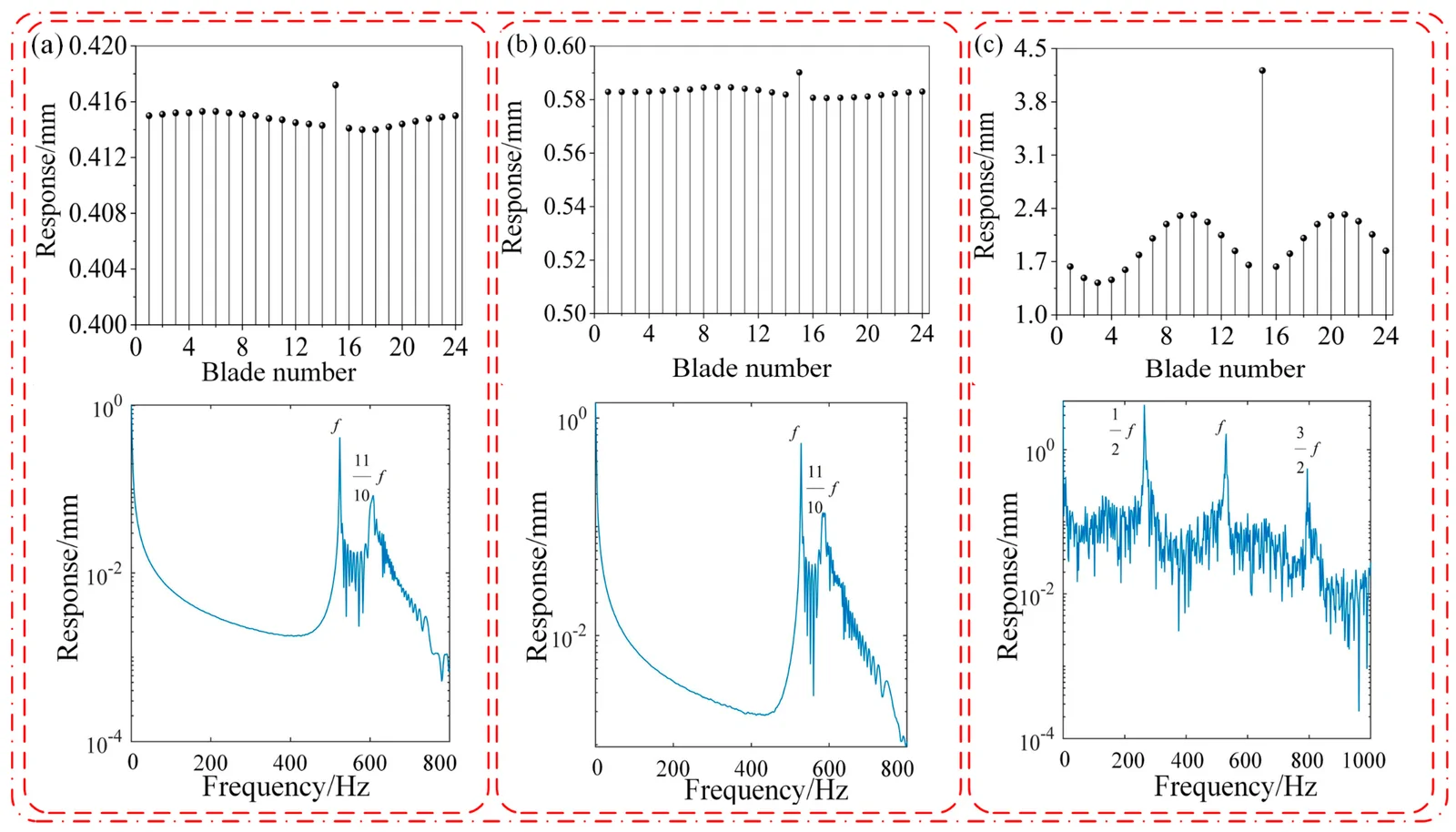
Blade amplitudes and tip response of Blade 15# for cracked blisk: (**a**) 15°, (**b**) 30°, and (**c**) 45°.

In this section, the effect of the blade angle on the response characteristics of the blisk with breathing cracks is examined. The blade−tip displacement amplitudes are evaluated under variable rotational speed conditions for blade angles of 15°, 30°, and 45°, based on the axial responses extracted at the blade−tip measurement points. The amplitudes of all 24 blades are compared in Figure 24a–c, which clearly indicate a dependence on blade angle. The corresponding vibration response spectra are also presented in the same figures. The 45° configuration produces the largest amplitudes, exceeding those of the 15° and 30° cases by more than three times; notably, the cracked blade exhibits a response amplitude approximately seven times higher than in the other configurations. The amplitudes of the remaining blades are nearly identical for the 15° and 30° cases, whereas in the 45° case, they display a periodic decrease–increase pattern along the circumferential direction (from blade 1# to 24#).

As illustrated in Figure 24a–c, the frequency response spectra exhibit increasingly pronounced nonlinear features with increasing blade angle. For the 15° case, a super−harmonic component at 1.1 times the excitation frequency (576.29 Hz) is observed. The 30° case is primarily dominated by the excitation frequency (528.07 Hz). In contrast, the 45° configuration exhibits strong nonlinear behavior, characterized by both sub−harmonic (1/2, 264.95 Hz) and super−harmonic (3/2, 794.85 Hz) components. Comparison among these cases indicates that increasing blade angle leads to a shift in the response peak toward higher frequencies, as well as a significant broadening of the response bandwidth. The 45° case exhibits the widest frequency band and multiple spectral peaks, reflecting enhanced nonlinear effects and more pronounced response localization.

### 4.4. Discussion

Cracks can induce complex dynamic behaviors and distinctive vibration characteristics in blisk structures, including super−harmonic and sub−harmonic resonance responses, vibration alterations arising from coupling between cracks and other nonlinearities, time−varying contact states associated with crack breathing, and severe vibration localisation. Even for minor cracks, super−harmonic and sub−harmonic resonances are readily excited, and these unique signatures have been widely utilized for crack localisation and depth estimation [29,31,32,33]. To fully elucidate the nonlinear vibration mechanism induced by cracks, it is essential to examine not only the resulting changes in natural characteristics and nonlinear response behavior but also the time−dependent evolution of crack−face contact during the breathing process [32,34]. For blisk assemblies, both structural mistuning and crack defects are known to cause intense localisation of forced vibration responses [21]. The present study further reveals that simultaneous consideration of crack breathing and blade geometric twist intensifies disk–blade coupling through crack–face contact nonlinearity, thereby exciting more pronounced sub−harmonic and super−harmonic resonances. This finding extends the existing understanding of geometrically induced high−order harmonic generation [8] and enriches the mechanistic framework linking geometric parameters with crack−induced nonlinearities in high−order harmonic vibrations.

## 5. Conclusions

This study systematically investigates the effects of breathing crack distribution and blade angle on the natural and dynamic response characteristics of an aero−engine blisk. The main findings are summarized as follows:

(1) The influence of crack distribution and blade angle on frequency−veering and modal localization is examined. Modes in the vicinity of the frequency−veering region are found to be particularly susceptible to localization. Under different crack patterns, either one or a few blades exhibit significant localization factors. Moreover, the blade angle markedly affects localization behavior, with the most severe modal localization occurring at a blade angle of 45°.
(2) The response characteristics under varying crack distributions and blade angles are evaluated. The configuration with two intact blades between cracks produces the largest response amplitudes. The introduction of breathing cracks not only shifts the response peaks toward lower frequencies but also broadens the frequency bandwidth and generates multiple secondary peaks, indicating enhanced nonlinearity and intensified response localization. Notably, the 45° blade angle case displays the strongest nonlinear response and the most pronounced localization.
(3) Resonance−based vibration tests are conducted on both intact and cracked integral blisk specimens. Comparisons of acceleration responses and waterfall plots under different excitation levels reveal that crack initiation leads to reduced natural frequencies and elevated response amplitudes. The experimental results are in good agreement with the numerical predictions, thereby validating the proposed modeling approach.
(4) The strong nonlinear response observed at a 45° setting angle is primarily attributed to the coupled vibration of the blisk. Given that actual aero−engine compressor and fan blades typically operate with twist angles ranging from 30° to 60°, these findings demonstrate that multiple−harmonic resonances induced by geometric twist angles must be explicitly incorporated into high−cycle fatigue assessments, providing critical engineering guidance for resonance avoidance design and fatigue life evaluation of twisted blisks.

## Figures and Tables

**Table 1:** Parameter settings of FEM and contact modeling.

Setting Name	Setting Value
Mode Type	3D Solid Model
Contact Type	Frictional and Bonded
Contact Algorithm	Augmented Lagrange
Automatic Time Stepping	On
Large Deflection	On

**Table 2:** Main structural parameters of the blisk.

Inner Diameter	Outer Diameter	Blade Angle β	Blade Length L	Blade Width H
100 mm	320 mm	30°	148 mm	50 mm

**Table 3:** Characteristics and classifications of representative modes of the blisk.

Mode Order	2	3	5	31	36
Pitch Diameter	1	2	3	2	0
Mode Family	M1	M1	M1	M2	M2
In Veering Region	No	Yes	Yes	Yes	No

## Data Availability

The data that support the findings of this study are available from the corresponding authors upon reasonable request.
